# Telehealth exercise for continence after gynaecological cancer treatment (TELE-CONNECT): a protocol for a co-designed pragmatic randomised controlled trial

**DOI:** 10.1186/s12905-024-03365-9

**Published:** 2024-09-27

**Authors:** Helena C Frawley, Kim Bennell, Rachel K. Nelligan, Angela Ravi, Nipuni Susanto, Simon Hyde, Orla McNally, Shih-Ern Yao, Karen E Lamb, Peixuan Li, Linda Denehy, on behalf of the TELE-CONNECT study team, Mark Merolli, Tom Jobling, Jennifer Kruger, Martha Hickey, Helen Brown, Lesley McQuire, Rowan Cockerell

**Affiliations:** 1 Melbourne School of Health Sciences, The University of Melbourne, 161 Barry Street, Parkville, VIC 3010 Australia; 2 Allied Health Research, The Royal Women’s Hospital Melbourne, 20 Flemington Rd, Parkville, VIC 3052 Australia; 3 Allied Health Research, Mercy Hospital for Women, 163 Studley Rd, Heidelberg, VIC 3084 Australia; 4 Centre for Health, Exercise and Sports Medicine, Department of Physiotherapy, The University of Melbourne, 161 Barry Street, Parkville, VIC 3010 Australia; 5 Department of Gynaecological Oncology, Mercy Hospital for Women, 163 Studley Rd, Heidelberg, VIC 3084 Australia; 6 Department of Obstetrics, Gynaecology and Newborn Health, University of Melbourne, 161 Barry Street, Parkville, VIC 3010 Australia; 7 Department of Gynaecological Oncology, The Royal Women’s Hospital, 20 Flemington Rd, Parkville, VIC 3052 Australia; 8 Department of Gynaecologic Oncology, Monash Health – Moorabbin Hospital, 823 – 865 Centre Road, Bentleigh East, 3165 VIC Australia; 9 Centre for Epidemiology and Biostatistics, Melbourne School of Population and Global Health, The University of Melbourne, 207 Bouverie St, Carlton, VIC 3053 Australia; 10 MISCH (Methods and Implementation Support for Clinical Health) research Hub, Faculty of Medicine, Dentistry and Health Sciences, The University of Melbourne, 207 Bouverie St, Carlton, VIC 3053 Australia; 11 Health Services Research, The Peter MacCallum Cancer Centre, 305 Grattan St, Melbourne, VIC 3052 Australia; 12 Department of Physiotherapy, The University of Melbourne, 161 Barry Street, Parkville, VIC 3010 Australia

**Keywords:** Gynaecological cancer, Urinary incontinence, Pelvic floor muscle training, Biofeedback, Telehealth, Physiotherapy, Randomised controlled trial

## Abstract

**Background:**

Urinary incontinence (UI) is the most prevalent pelvic floor disorder following treatment for gynaecological cancer with a distressing impact on quality-of-life in survivors. Physiotherapist-supervised pelvic floor muscle (PFM) training is recommended as the first-line intervention for UI in community-dwelling women. However, it is not known if this intervention is effective in women following treatment for gynaecological cancer, nor whether PFM training can be delivered entirely remotely. The primary aim of this study is to investigate if a telehealth-delivered PFM training program incorporating a novel biofeedback device reduces UI compared with usual care, following gynaecological cancer.

**Methods:**

This is a pragmatic, two-arm parallel-group, stratified superiority randomised controlled trial recruiting 72 participants (ACTRN12622000580774). Recruitment sites include gynaecology-oncology outpatient clinics, supplemented by advertisements through community foundations/social media/care groups. Participants must have completed primary cancer treatment at least 6 months prior or adjuvant therapy at least 3 months prior, for Stage I, II or III uterine, cervical, fallopian tube, primary peritoneal or ovarian cancer or borderline ovarian tumour, and have UI occurring at least weekly. Participants randomised to the usual care group will receive bladder and bowel advice handouts and one audio telehealth physiotherapist consultation to answer any queries about the handouts. Participants randomised to the intervention group will receive the same handouts plus eight video telehealth physiotherapist consultations for PFM training with a biofeedback device (femfit^®^), alongside a home-based program over 16 weeks. The primary outcome measure is a patient-reported outcome of UI frequency, amount and interference with everyday life (measured using the International Consultation on Incontinence Questionnaire – Urinary Incontinence Short Form), immediately post-intervention compared with baseline. Secondary outcomes include quality-of-life measures, bother of pelvic floor symptoms, leakage episodes, use of continence pads and global impression of change. We will also investigate if the intervention improves intra-vaginal resting and squeeze pressure in women in the intervention arm, using data from the biofeedback device.

**Discussion:**

If clinical effectiveness of telehealth-delivered physiotherapist-supervised PFM training, supplemented with home biofeedback is shown, this will allow this therapy to enter pathways of care, and provide an evidence-based option for treatment of post-cancer UI not currently available.

**Trial registration:**

Australian New Zealand Clinical Trials Registry (ANZCTR), ID 12622000580774. Registered 20 April 2022.

**Supplementary Information:**

The online version contains supplementary material available at 10.1186/s12905-024-03365-9.

## Background

 Urinary incontinence (UI) is the most prevalent type of pelvic floor disorder, with prevalence estimates ranging from 25 to 45%, depending on population, study methods, measures, UI definition, and disclosure rates [1]. Incontinence negatively impacts on a woman’s social and emotional wellbeing and ability to engage with the community, with 39% of women who experience UI reporting that they are less confident in leaving the house, 32% suggesting that it affects their mental health and wellbeing and 25% indicating that it affects their relationships with family and friends [2]. The majority of women do not disclose UI nor seek help, due to stigma, the intimate nature of their symptoms and the perception of UI as being normal [2], yet UI significantly diminishes a woman’s quality-of-life (QoL) [3].

Prevalence of pelvic floor disorders appears higher in women following treatment for gynaecological cancer, with a systematic review identifying the prevalence of UI at 76% following cervical cancer and 84% following uterine cancer [4]. A recent cohort study found women who received adjuvant therapy following surgery for gynaecological cancer were nearly five times more likely to develop moderate-to-very severe UI three months after surgery compared with surgery only [5]. While incidence rates of gynaecological cancer continue to rise, survival rates are improving [6], hence QoL in the recovery and survivorship phase following cancer treatments is important to address. In addition to the prevalence, the impact of UI is higher in gynaecological cancer survivors compared to the general population [7] and this burden is likely to negatively impact survivor’s ability to re-engage with life post-cancer treatments. Despite these impacts, screening for pelvic floor disorders following cancer treatments, especially in high-risk cohorts, and onward referral are not routine. A recent qualitative study identified that both clinicians and patients are aware of the burden of pelvic floor disorders in this population and agree there is a need to better manage this burden [8].

Reasons for lack of clear guidelines to address pelvic floor disorders in this population may be insufficient knowledge of the mechanisms by which cancer treatments impact the pelvic floor and insufficient evidence to inform care. Evidence to support pelvic floor muscle (PFM) training as a low-risk, low-cost exercise therapy for women with UI is well established [9]. Supervised, intensive PFM training changes muscle physiology and morphometry [10]. Women with stress UI (the most prevalent type of incontinence) who undertook PFM training were six times more likely to report cure or improvement (74% versus 11%; RR 6.33, 95% CI 3.88 to 10.33) than controls, and women with any kind of UI who perform PFM are twice as likely to report cure or improvement than control groups (67% versus 29%; RR 2.39, 95% CI 1.64 to 3.47) [9]. However, due to potential effects of gynaecological cancer and its treatment (surgery, radiotherapy and chemotherapy) on pelvic floor structures [11–13], gynaecological cancer survivors may respond differently to PFM training compared to non-cancer populations and may require more intensive or longer duration training in order to achieve the same improvement as the non-cancer population. Therefore, population-specific investigation is required. Our systematic review of randomised controlled trials (RCTs) to investigate the effectiveness of PFM therapies to treat UI following gynaecological cancer identified no evidence of improvement from two small studies with under-dosed interventions [14]. Our recent update of this review identified one new RCT that indicated an improvement in urogenital distress and UI impact [15]. However, no sample size calculation was reported, and aspects of the intervention were unclear. Therefore, evidence to inform practice is weak. Due to the impact of UI, improving QoL following gynaecological cancer treatments is an identified priority [7, 16–18]. Effective treatments that provide equity of access in a format that is acceptable to women with UI following gynaecological cancer are required.

Telehealth-delivered pelvic physiotherapy provides clinicians and patients with the option of remote health care assessment and treatment. This overcomes challenges imposed by the COVID-19 pandemic (restrictions to on-site access and provision of health care services), the lack of physiotherapists with specialist knowledge outside of metropolitan centres, and in some regions, lack of patient access to specialised health care centres due to long distances. Systematic reviews suggest PFM training or education delivered by telemedicine and digital technologies may improve UI [19–21]. However, no RCT has tested an entirely remotely-delivered treatment programme supplemented by a patient home biofeedback device to confirm correctness of PFM contraction, nor any type of telehealth-delivered PFM program to women following treatment for gynaecological cancer. Our group recently conducted the first feasibility trial combining these elements, to treat urinary or faecal incontinence in 32 women following treatment for gynaecological cancer [22]. This study established the feasibility of this method, but without evidence of clinical benefit. A fully-powered pragmatic RCT with potential for rapid clinical implementation [23] is now required. The primary aim of this study is to test the hypothesis that a physiotherapist telehealth-delivered PFM training program incorporating a novel biofeedback device will reduce UI at 17 weeks after intervention compared with usual care, in patients with UI following gynaecological cancer.

## Methods

### Trial design

This is a pragmatic, two-arm parallel-group, stratified, superiority randomised controlled trial, to investigate the clinical effectiveness of the telehealth-delivered PFM training to treat UI in women following gynaecological cancer, called TELE-CONNECT: TELehealth Exercise for CONtinence after GyNaEcological Cancer Treatment. Reporting will comply with CONSORT and appropriate extensions [24–26] and the Consensus on Exercise Reporting Template for Pelvic Floor Muscle Training (CERT-PFMT) guidelines [27]. The trial was prospectively registered (20.04.2022) with the Australian New Zealand Clinical Trials Registry (ACTRN12622000580774). The trial is sponsored by the University of Melbourne and the lead investigator will respond to any auditing requirements requested by the sponsor. Multi-site ethics approval has been obtained from the Human Research Ethics Committee of Monash Health (RES-21-0000-626 A). The current study protocol (version 8) and participant information form (available online as Additional Files 1 and 2) and any protocol amendments will be detailed in the trial registration following ethics approval. A Data Monitoring Committee will not be required due to the low-risk nature of the intervention. However, a Trial Advisory and Safety Committee will be established to oversee progress and processes of the trial, to discuss any reported adverse events (AE) that may arise and to ensure the consumer perspective is represented.

### Recruitment

Recruitment will be via targeted invitations to potentially eligible women (see Table 1) from public outpatient gynae-oncology clinics of participating health services (*n* = 3). Potentially eligible women (those receiving treatment for histologically confirmed uterine, endometrial, cervical, fallopian tube, primary peritoneal or ovarian tumour or borderline ovarian tumour, International Federation of Gynecology and Obstetrics (FIGO) cancer staging system classification stages I-III) will be identified and approached to participate by staff working at the relevant health services. We will also recruit women from the community by inviting patients on existing research databases who have consented to be involved in future research to participate; via the private rooms of participating gynaecologists, and via advertisements through community foundations/social media/care group advertisements. Women who are potentially eligible will receive a follow up call from the trial coordinators to discuss the study in greater detail and confirm full eligibility. We will aim to recruit 36 participants per treatment arm; therefore 72 participants will be recruited in total.

### Participants

The eligibility criteria in Table 1 will be used to screen participants for this study.

**Table 1:** Eligibility

**Inclusion Criteria**	**Exclusion Criteria**
• Women following cancer treatment (with and without radiotherapy) for Stage I, II or III uterine, cervical, fallopian tube, primary peritoneal or ovarian cancer or borderline ovarian tumour • Primary cancer treatment completed ≥ 6 months ago or adjuvant therapy completed ≥ 3 months ago • Self-reported urinary incontinence (≥ 1 episode per week for last 4 weeks) • Aged 18 years or above • Have a home internet connection and a smartphone • Ability to speak and read English sufficiently for purposes of the study	• Have received > 1 physiotherapist-supervised pelvic floor treatment for urinary incontinence (in clinic or telehealth) since commencing cancer treatment or in the previous 4 years (whichever is most recent) • Have had pelvic surgery for incontinence or pelvic organ prolapse in the last 2 years • Have a significant neurological disorder • Have a severe physical or psychiatric impairment • Have a severe pelvic organ prolapse • Have a vaginal pessary ring in situ • Pregnant or breastfeeding • Have given birth within the last 12 months • Not willing to use an intra-vaginal biofeedback device or videoconferencing software • Unable to give informed consent or complete all study and assessment procedures

### Procedure

**Fig. 1:**
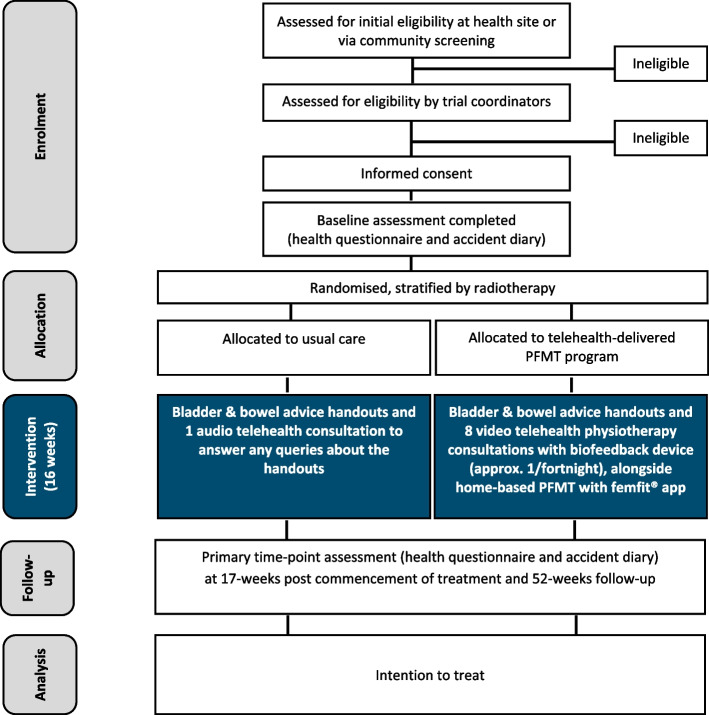
Flow diagram of the study phases. PFMT=pelvic floor muscle training

 The trial phases are outlined in Fig. 1. Screening information and study consent forms will be stored within REDCap and accessible only by password to the trial coordinators. Following the consent process, participants will be enrolled into the study. Participants will then be asked to complete an accident diary and author-designed health questionnaire at baseline via a web-based platform (REDCap) or in hard copy (if preferred by participant). If hard copies are completed, responses will be entered into the study computer database by a trial coordinator. Once returned, baseline material will be checked by the trial coordinators for completeness and to confirm relevant eligibility criteria are still met. Each participant will receive a unique study ID code, and this will be documented in the participant’s record/database in addition to all study documents.

### Data collection and management

Individually identifiable information will be accessed in the form of medical records of patients who consent to be contacted for this study in order to confirm potential eligibility. Medical records of all patients who consent to participate and complete baseline questionnaires will also be reviewed to confirm cancer-related medical history including tumour site and stage. Telephone contact details may also be obtained from patient medical records by a health professional member of the patient’s treating team and provided to the trial coordinators by this person. Patient contact details will only be collected following consent to contact being obtained from the patient. This information will be saved in a password-protected electronic document, stored electronically within secure password-protected servers. Questionnaires may be completed on paper or electronically and will only contain participant study codes. Paper copies will be stored in locked filing cabinets, separate from a cabinet containing any identifiable data and only accessible to the trial coordinators. Electronic copies will be stored in the REDCap website, accessible only to the trial coordinators by password protection. Data from within REDCap will be exported to Microsoft Excel and other statistical packages used by the researchers for analyses. These will be stored securely on password-protected servers. Intra-vaginal pressure profile data from the femfit^®^ biofeedback devices supplied to participants in the intervention arm will be exported from the femfit^®^ server for analysis, accessible to trial coordinators, study physiotherapists and statisticians.

Trial coordinators will encourage participants to complete questionnaires in full and return/upload by due date. Reminder communication will be via email, phone text message or voice calls. If participants fail to respond to three reminder communications, their data will be considered missing for that follow-up time-point. If a participant who has provided consent fails to return baseline questionnaires following reminders, they will be considered lost to recruitment.

### Randomisation and allocation concealment

Eligible participants will be randomised (in permuted random blocks stratified by radiotherapy or not) into one of two groups with a 1:1 allocation ratio. Randomisation will be concealed using the REDCap randomisation module. The randomisation schedule will be computer-generated by an independent statistician to ensure study statisticians remain blinded to treatment allocation and stored on a password-protected website (REDCap) at the University of Melbourne, maintained by a researcher not involved in either participant recruitment or administration of primary/secondary outcome measures.

Group allocation will be revealed by a different member of the research team, who has had no contact with participants. If randomised to intervention, each woman will be further randomly allocated to one of the study physiotherapists, according to a randomisation schedule supplied by the statistician. As this is a pragmatic trial and as is common to exercise interventions, participants will not be blinded to group allocation. Therefore, as the primary and secondary outcomes are participant-reported, by default the assessors of these outcomes (the participants) are not blinded. The statistical analysis plan will be developed by a biostatistician blinded to group allocation and made publicly available prior to unblinding and statistical analysis.

### Interventions

#### Usual care

Participants in this group will receive bladder and bowel advice handouts currently available from the Continence Foundation Australia and the Australian Government Department of Health. A trial coordinator will send the handouts via email or post (dependent on the participant’s preference), directly after randomisation. After randomisation a trial coordinator will schedule one, (up to) 15-minute tele-health consultation (via telephone) with participants, 1–2 weeks after completion of baseline assessments. During this telephone call, the researcher will check the handouts have been received, answer any handout or study related queries and remind the participant of the dates of their follow-up assessments. The researcher will not provide any tailored PFM advice/instruction.

### Telehealth-delivered PFM training

**Fig. 2:**
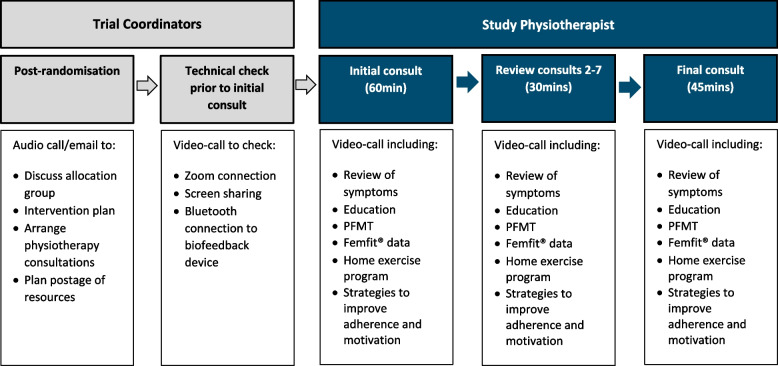
Participant flow following randomisation to telehealth-delivered PFM training group

 Participants in the intervention group will receive the same handouts as the control plus eight physiotherapist-supervised telehealth-delivered consultations within a 16-week period (see Fig. 2).

Pelvic floor muscle exercise will be supported by a Therapeutic Goods Administration (TGA) approved femfit^®^ biofeedback device [28]. Reliability and validity of this device has been demonstrated [29]. Participants will be sent their own device and instructions to download the femfit^®^ app to their mobile phone which will allow them to visualise their PFM contraction in real-time, provide them with the home exercise program, and track their adherence to the program. The device is an intra-vaginal pressure sensor array which measures resting and squeeze pressure (mmHg) along the length of the vagina. Data are transmitted to a mobile device (Android or ios) via Bluetooth for real-time visualisation during exercise. The sensor array is encased in a soft, medical grade silicone which is 80 mm long, 24 mm wide and 4 mm thick. The flexible sensor is inserted into the vagina and does not distend the vaginal space. The eight pressure sensors record intra-vaginal pressure detected along the vaginal length which are displayed to the participant on their mobile phone via the device app. The treating physiotherapist will view the participant’s pressure profile with PFM contraction and relaxation in real-time, thereby allowing remote teaching of a correct pelvic floor muscle contraction [30].

We will aim for the initial consultation to be within 2 weeks of baseline assessments. Subsequent tele-health appointments will be made by the treating study physiotherapist in consultation with the participant. Prior to a participant’s first physiotherapist consultation, a trial coordinator will schedule and conduct a teleconference consultation with the participant to practise the technical aspects of internet connection and screen sharing (approximately 15–30 min). Study physiotherapists will be provided a copy of each baseline questionnaire of participants allocated to them. This will be sent by a trial coordinator via email and prior to a participant’s initial consultation. This is to facilitate discussions between the physiotherapist and participants about past medical history and current symptoms at the first consultation, and to save participant time and burden explaining history and symptoms already outlined in the baseline questionnaire.

### Study physiotherapists

We will recruit physiotherapists from our study sites and/or the broader community to deliver the tele-health consultations. Physiotherapists will be eligible if they (i) have postgraduate training in pelvic floor physiotherapy; (ii) are able to commit to accepting at least one new study participant for their per-protocol intervention each week; (iii) agree to comply with all study processes and procedures. Study physiotherapists will receive a detailed study manual and will complete training in study processes and procedures (see Table 2). Within the study manual, a ‘consultation notes’ template will be included and used to document each participant consultation. Physiotherapists can choose to complete consultation notes electronically.

**Table 2:** Components of the physiotherapist-supervised tele-health consultations

CERT-PFMT item	Description of the TELE-CONNECT intervention
1. Exercise equipment	The femfit^®^ biofeedback device to help teach the correct PFM contraction technique, to monitor PFM strength gains and facilitate PFMT adherence.
2. Instructor qualifications	All physiotherapists providing the intervention will have postgraduate training in pelvic floor physiotherapy. Specific study training will include: - PFM assessment and exercise instruction processes; - instructions for teaching use of the biofeedback device; - simulated video-consults to practise video-consultation skills.
3. Individual or group	Individual physiotherapist supervised telehealth-delivered instruction in PFMT and individual home-based PFMT.
4. Supervised or not supervised	Exercise supervision will be provided remotely by a trained physiotherapist via 8 telehealth-delivered consultations, over 16 weeks. Between tele-health consultations participants will complete their PFMT unsupervised.
5. Adherence	Adherence to PFMT will be reported within the femfit^®^ app. Attendance at physiotherapy consultations will be recorded by the physiotherapist.
6. Motivation ^a^	Biofeedback will be provided via the femfit^®^ device and associated app. An extensive list of motivational strategies including goal setting, specific instructions/wording on how to perform PFMT verbal persuasion, visual cues that may be suggested by the physiotherapist to aide PFMT will be implemented.
7. Progression decision rules ^a^	The femfit ^®^ app’s pre-determined, exercise program and recommended progressions will be used which are evidence-based and clinically validated (28). Within the femfit^®^ app the intensity and repetitions of the exercises increase every 4 weeks. In the first month, the exercise program will take around 8 min to complete daily. This increases to 12 min in the second month, 16 min in months 3 and 4 and for the remainder (maintenance phase). The position to do the exercises also changes from lying to supported standing, to standing.
8. Exercise description	PFM exercises included in the femfit ^®^ app are: maximal contraction, co-ordination, endurance, contraction with cough. These are described in the femfit^®^ app as “Squeeze”, “Rapid”, “Enduro”, and “Knack”.
9. Home program	PFM exercises, as prescribed by the physiotherapist, will be conducted at home. Specific home exercise details regarding position, time, dosage, effort and use of the femfit^®^ app will be provided by the physiotherapist at each consultation.
10. Non-exercise components ^a^	Intervention participants will receive the same informational handouts regarding good bladder and bowel health as the control group. Additional information will be provided as indicated: - For urinary urgency and frequency or urge urinary incontinence: bladder training including urinary urge suppression techniques, education on fluid intake modification including caffeine reduction if relevant, and education in good bladder and bowel habits. - For faecal incontinence: bowel routine training, education on dietary fibre including psyllium supplementation, education on faecal urgency suppression techniques.
11. Adverse events	Explicit descriptions of adverse events will be reported
12. Setting	Tele-health and home exercise programs will be conducted in participant’s home.
13. Dosage	Refer to details provided in Item 8. Exercise description
14. Generic or tailored ^a^	The femfit^®^ app’s pre-determined, exercise program will be used which is evidence-based and clinically validated (28). Additional tailoring to each participant may be recommended by the treating study physiotherapist based on assessment findings.
15. Starting level assessment ^a^	The femfit^®^ app’s pre-determined starting level will be used. The starting level may be modified by the treating study physiotherapist based on assessment findings.
16. Adherence to intervention ^a^	A trial coordinator will review and analyse consultation notes and conduct audits of the audio recorded consultations to assess the physiotherapists’ adherence to trial protocols.

*PFM* Pelvic floor muscle, *PFMT* Pelvic floor muscle training

^a^ Preliminary research: Prior to this trial, a pilot study was conducted including consultation and co-design of resources and intervention processes and procedures with the recruited study physiotherapists and participants who have had gynaecological cancer treatment. All study materials have been created specifically for this intervention

### Treatment fidelity

The trial shall ensure delivery of treatment fidelity: treatment integrity and treatment differentiation [31]. Throughout the trial, clinicians will be encouraged to discuss any queries that arise while delivering the interventions with the researchers. Treatment notes from consultations will be assessed for physiotherapist adherence to trial protocol. In addition, fidelity of treatment receipt will be provided via data obtained from the femfit^®^ app, which will inform how well the participants adhered to the prescribed exercise protocol.

### Outcome measures

Table 3 summarises the data collected, the trial outcome measures and assessment time-point for each data point and outcome measure. The primary outcome is participant-reported UI, measured on the International Consultation on Incontinence Questionnaire – Urinary Incontinence Short Form (ICIQ-UI SF) [32]. The ICIQ-UI SF is a 3-item self-reported questionnaire capturing frequency, severity and bother of any type of UI in the past 4 weeks. It is scored by summing the responses to these 3-items to create one overall score ranging from 0 to 21, with higher scores indicating greater impact of UI. It is a Grade A validated measure of patient-reported UI that has been recommended for use in UI clinical trials [33] and has established responsiveness in a female population with UI, over an 8-week PFM training intervention [34]. Conclusions regarding effectiveness of the intervention will be based on the 17-week change in this outcome. Secondary outcome measures will investigate generic and condition-specific health-related QoL, bother of pelvic floor symptoms, leakage episodes, use of continence pads, global impression of change, acceptance and use of technology and physical activity. Changes in PFM resting and contraction pressure in women in the intervention arm from baseline to 17 weeks will be assessed using data from the biofeedback device (see Table 3).

**Table 3:** Outcome summary (follow-up time-points are relative to commencement of treatment)

Domain	Instrument	Timepoint		
		T_0_: Baseline	T_1_: 17 weeks	T_2_: 52 weeks
**Descriptive measures** ^a^				
Age, height, weight	Self-reported within health questionnaire	✔		
Geographical location	Self-reported within health questionnaire	✔		
Current employment status	Self-reported within health questionnaire	✔		
Current relationship status	Self-reported within health questionnaire	✔		
Hormone status	Self-reported within health questionnaire	✔		
Birth history	Self-reported within health questionnaire	✔		
Comorbidities	Self-reported within health questionnaire	✔		
Pre-cancer treatment presence of incontinence	Self-reported within health questionnaire	✔		
Cancer-specific medical history - Stage of cancer - Cancer location - Past cancer treatment - Time since last cancer treatment - Use of intra-vaginal dilators	Self-reported within health questionnaire	✔		
Cancer treatment since commencing this study	Self-reported within health questionnaire		✔	✔
Health changes since commencing this study	Self-reported within health questionnaire		✔	✔
Current pelvic floor muscle exercise levels	Self-reported within health questionnaire	✔	✔	✔
Medications relevant to pelvic floor function	Self-reported within health questionnaire	✔	✔	✔
**Primary outcome**				
Urinary incontinence	ICIQ-UI SF	✔	✔	✔
**Secondary outcomes**				
Condition-specific quality-of-life	ICIQ-LUTSqol	✔	✔	✔
Bother of pelvic floor symptoms	PFBQ	✔	✔	✔
Acceptance and use of technology	UTAUT-II	✔	✔	
Health-related quality-of-life	EQ-5D-5 L questionnaire	✔	✔	✔
Number and severity of leakage episodes	Self-reported within accident diary	✔	✔	✔
Number of leakage episodes by provocation	Self-reported within accident diary	✔	✔	✔
Number of continence pads used	Self-reported within accident diary	✔	✔	✔
Physical activity	IPAQ short form	✔	✔	✔
Global Impression of Change	PGIC		✔	✔
Treatment satisfaction	7-point ordinal scale		✔	✔
**Harms**				
Adverse Events	Reported by participants or clinicians		✔	✔
**Other Measures** (intervention group only)				
PFM pressure changes (rest, contraction)	Data from femfit^®^ device	✔	✔	
Adherence:				
- To attendance at physiotherapy video-consultations	Physiotherapist's consultation documentation		✔	
- To exercise protocol	Data from femfit^®^ device		✔	

*ICIQ-UI SF* International Consultation on Incontinence Questionnaire – Urinary Incontinence Short Form [32], *ICIQ-LUTSqol* International Consultation on Incontinence Questionnaire – Lower Urinary Tract Symptoms Quality of Life [35], *PFBQ* Pelvic Floor Bother Questionnaire [36], *UTAUT-II* Unified theory of acceptance and use of technology [37], *PGIC* Patient Global Impression of Change [38], *IPAQ* International Physical Activity Questionnaire Short Form [39]

^a^All descriptive measures are self-reported within an author-designed health questionnaire

### Health economic data

We will collect data to help inform a health-economic or cost-effectiveness analysis, in the case that clinical effectiveness is shown, and this may be published in a separate paper. Data will include cost of physiotherapist’s time to provide the intervention, cost of resources to provide the intervention, cost of pads for bladder leakage, laundry costs associated with UI, health service use related to UI and work productivity.

### Ancillary and post-trial care

The intervention being tested in this trial, PFM training, is considered a low-risk conservative therapy. However, in recognition of the vulnerability of the population and condition (patients who have undergone treatment for gynaecological cancer) the study lead and trial coordinators will make every effort to support participants who express any distress as part of their involvement in the trial. We will offer any participant who becomes distressed, the option to pause or cease their involvement in the study, and we will provide details of community services for mental health support. For participants in the intervention group who require a short break or to reschedule their appointment to another time, we will accommodate this request. Participants who complete their final assessment (week 52) will receive an AUD $20 gift card as a token of appreciation for their involvement in the study. Post-trial, we will provide all participants with information regarding community available continence resources, should they require this.

### Adverse events

Due to the nature of the intervention, any risks to participants are likely to be minor and transient. The reporting and handling of all AE will be in accordance with National Health and Medical Research Council guidelines [40], which defines AE as: “Any untoward medical occurrence in a patient or clinical trial participant administered a medicinal product and that does not necessarily have a causal relationship with this treatment” and serious AE as any AE that: results in death; is life threatening; requires inpatient hospitalisation or prolongation of existing hospitalisation; results in persistent or significant disability/incapacity; is a congenital anomaly/birth defect. Due to the low-risk nature of the interventions in this trial, serious AE are extremely unlikely but will be reported to the primary Human Research Ethics Committee should they occur. We will record all AE, including minor, expected or unexpected (as described in the PICF), according to the National Cancer Institute Common Terminology Criteria for Adverse Events (NCI-CTCAE) v5 criteria [41].

Information regarding AE may be reported by participants in the 17 and 52-week follow-up assessment questionnaires. Adverse events may also be reported by the participant directly to the treating study physiotherapist or to the trial coordinators. Any AE reported to the study physiotherapist will be recorded in the consultation notes and if serious will be reported to the PI and entered into the REDCap data base according to the NCI Guidelines for Investigators reporting attribution and severity [42]. All AEs will be reported to the internal Trial Advisory and Safety Committee who will be responsible for deciding what action if any is needed on a case-by-case basis.

### Sample size

A total of 72 women (36 per group) is required to detect a minimum clinically important difference (MCID) of 2.5-units in change in the ICIQ-UI SF (primary outcome) between baseline and 17 weeks (primary time point) between the two groups. This assumes 80% power, a two-tailed significance level of 5%, equal standard deviation (3.2 units) in each group [34], a conservative correlation of 0.4 between baseline and 17-week scores, an intra-cluster correlation of 0.05 with 3 physiotherapists treating approximately 10 patients each [43] (to account for potential clustering by physiotherapists in the intervention arm), and 15% attrition, to ensure we will have primary outcome data from at least 85% of participants [44].

### Statistical analyses

All analyses will be described a priori in a detailed Statistical Analysis Plan and made publicly available while biostatisticians are blinded to treatment allocation. Demographic and baseline characteristics of participants will be summarised as appropriate (means and standard deviations for continuous variables that are distributed approximately symmetrically, medians and interquartile ranges for other continuous variables, counts and percentages for categorical variables). The main comparative analyses will be performed on an intention-to-treat basis, including all randomised participants in their allocated study group. The primary outcome (ICIQ-UI SF) will be analysed using a constrained longitudinal data analysis model [45], which will consist of all outcome scores (baseline, 17 weeks and 52 weeks) and the model including factors representing treatment group, time (categorical), and a group-by-time interaction, with the restriction of a common baseline mean across treatment groups. This restriction is due to the assumption that there are no differences in the mean outcome between the two groups at baseline (i.e., assuming effective randomisation). Models will include the stratification variable (radiotherapy) and random effects for physiotherapist (intervention arm only). These models provide valid inference in the presence of missing data if the data are missing at random. Standard diagnostic plots will be used to check model assumptions. The primary hypothesis will be evaluated by obtaining the estimated difference in mean change in the ICIQ-UI SF from baseline to 17-weeks post-commencement of treatment (primary time point) between the two intervention arms, two-sided 95% confidence interval and p-value. The treatment effect at 52 weeks post-randomisation (secondary time point) will be estimated to assess maintenance of treatment effect. In addition to the intention-to-treat effect, we will obtain the complier average causal effect by making use of collected adherence data [46].

Similar analyses will be undertaken for secondary continuous outcomes (ICIQ-LUTSqol, PFBQ, EQ-5D-5 L, UTAUT-II domains 1–9, METminutes (walking, moderate-intensity activities, vigorous-intensity activities). Binary secondary outcomes (PGIC and physical activity category) will be compared between groups separately using logistic regression, adjusting for the stratifying variable of radiotherapy, and fitted using generalised estimating equations to account for clustering, with results reported as risk ratios and risk differences. Poisson regression models fitted using generalised estimating equations will be used for secondary count outcomes (number of leakage episodes overall and by severity and provocation, number of continence pads used). The number and percentage of participants with adverse events will be summarised by intervention group. Measures which relate to the intervention group only –intra-vaginal resting and squeeze pressures – will be analysed as continuous outcomes using paired t-tests (mean difference and 95% confidence intervals) to determine if there are differences pre-post intervention. Descriptive statistics will be calculated to assess adherence (attendance at physiotherapy video-consultations and to exercise protocol) to the intervention.

The economic evaluation will have a societal perspective and will assess both the cost of the intervention at 17 weeks and the cost-effectiveness and cost-utility of the intervention group *versus* the control group at 52-weeks. Quality-adjusted life years (QALYs) gained [47] for the intervention compared to control at 52 weeks will be assessed. QALYs will be calculated based on utility scores using the EQ-5D-5 L [48, 49] at baseline and 52 weeks. QALYs will also be calculated using the ICIQ-LUTSqol [35], a condition specific quality of life measure, at baseline and 52 weeks. Analysis will be combined with the primary clinical outcome measure to establish the incremental cost-effectiveness ratio of the intervention. The difference in participant resource use related to incontinence (e.g. pad use, medication use, health service use) and productivity lost between baseline and 52 weeks will be compared for intervention and control groups. The association between utility gains on the EQ-5D-5 L and productivity will be compared between the intervention and control groups. The 52-week economic evaluation will be reported separately from the main trial and only if clinical effectiveness is shown.

### Timelines

Ethical approval was obtained from the Human Research Ethics Committee Monash Health on 09.12.2021. Recruitment commenced in August 2022 and is expected to be completed in September 2024. The trial is due for completion in September 2025 when all participants will have completed 12-month data.

### Patient and public involvement

Two consumers with lived experience of gynaecological cancer, and a community representative from the national peak body promoting bladder and bowel control health in Australia (Continence Foundation of Australia) have been active co-investigators in this study and members of the Trial Advisory and Safety Committee. These three consumers have been involved in the study co-design phase and consulted on all participant-facing documents and recruitment procedures. We anticipate their participation will continue through analysis and dissemination phases.

### Dissemination

The main trial will be published in an oncology or general medical journal. Statistical code may be made available from the statistician, upon request from individual researchers. Data may be made available from A/Professor Frawley, upon request from individual researchers. In addition, the results of the trial will be disseminated through avenues such as conference presentations, professional organisations, media, social media and consumer organisations.

## Discussion

This protocol has presented the background and rationale of why this study is needed, the protocol for a pragmatic, two-arm parallel-group stratified, superiority randomised controlled trial, to investigate the clinical effectiveness of the telehealth-delivered PFM training to treat UI in women following gynaecological cancer. Primary and secondary analyses – including the proportion that meet or exceed the minimal clinically important difference for the primary outcome [34] – will be included in the main results paper. Due to the paucity of evidence to date to guide clinical care of UI in this population, this trial will provide valuable data to inform clinical decision-making for women with residual or *de novo* UI following completion of primary cancer treatment. If clinical effectiveness of telehealth-delivered physiotherapist-supervised PFM training, supplemented with home biofeedback is shown, this will allow this therapy to enter pathways of care, and provide an evidence-based option for treatment of post-cancer UI not currently available.

## Supplementary Information

Additional file 1: HREC-approved study protocol: Effectiveness of telehealth-delivered pelvic floor muscle training incorporating novel biofeedback versus usual care, to treat incontinence in women following gynaecological cancer: a co-designed pragmatic RCT protocol (version 8).

Additional file 2: TELE-CONNECT study participant information form.

## Data Availability

No datasets were generated or analysed during the current study.
