# Supplementary material for: Telehealth exercise for continence after gynaecological cancer treatment (TELE-CONNECT): a protocol for a co-designed pragmatic randomised controlled trial
Source: BMC Womens Health. 2024 Sep 27;24:529. doi: 10.1186/s12905-024-03365-9 (PMC11430120; doi:10.1186/s12905-024-03365-9)
Supplement: Supplementary file 1 — Additional file 1: HREC-approved study protocol: Effectiveness of telehealth-delivered pelvic floor muscle training incorporating novel biofeedback versus usual care, to treat incontinence in women following gynaecological cancer: a co-designed pragmatic RCT protocol (version 8). [file 12905_2024_3365_MOESM1_ESM.pdf]

Effectiveness of telehealth-delivered pelvic floor muscle training incorporating novel biofeedback versus usual care, to treat incontinence in women following gynaecological cancer: a co-designed pragmatic RCT protocol

Project Team
A/Prof Helena Frawley
Prof Linda Denehy
Prof Kim Bennell
Dr Rachel Nelligan
Angela Ravi
A/Prof Simon Hyde
A/Prof Orla McNally
Dr Shih-Ern Yao
A/Prof Karen Lamb
Fiona McManus
Nipuni Susanto
Dr Zobaida Edib
Peixuan Li

On behalf of the TELE-CONNECT Study Team
Dr Mark Merolli
Prof Tom Jobling
Prof Chantale Dumoulin
Dr Jennifer Kruger
Prof Martha Hickey
Ms Rowan Cockerell
Dr Helen Brown
Ms Lesley McQuire

Sponsor
University of Melbourne

CONFIDENTIAL

This document is confidential and the property of the University of Melbourne.
No part of it may be transmitted, reproduced, published, or used without prior written authorisation from the institution.

STATEMENT OF COMPLIANCE

This document is a protocol for a clinical research study. The study will be conducted in compliance with all stipulations of this protocol, the conditions of ethics committee approval, the NHMRC National Statement on Ethical Conduct in Human Research (2007) and the Note for Guidance on Good Clinical Practice (CPMP/ICH-135/95).

Document created:
2021/07/05

Modified:
2024/06/03
2023/06/01
2022/12/19
2022/11/21
2022/11/06

2022/05/02
2022/03/29
2021/11/25
2021/07/05

Details of Modification:

Date	Version	Modification
03.06.2024	8	Minor amendments for HREC<br>1. TELE-CONNECT_RCT Protocol v8 03.06.2024<br>a. New statistician, Peixuan Li, added as Associate Investigator<br>b. Change made to title prefix of associate investigator Lamb from Dr to A/Prof<br>c. Clarification made to primary timepoint where the 17-week questionnaire will be administered "17 weeks post *commencement of treatment"* not "17 weeks post *randomisation"*. Following sections of protocol changed to reflect this:<br>o Protocol synopsis "Study procedures" (p7)<br>o Protocol synopsis "Statistical Procedures: Sample Size Calculation & Analysis Plan" (p8)<br>o Section 12.2 Statistical Analysis Plan (p41)<br>d. Following changes made to table **4.6 - Primary and Secondary Outcome Measures** pp18-24<br>o Addition of "statistical analysis plan" column to table<br>o Addition of "severity" of leakage episodes, "Number of leakage episodes by provocation" rows to outcome measures collected from Accident diary. Description, Scale, Time-points measured and Statistical Analysis plan column headings filled in<br>o International Consultation on Incontinence Questionnaire Lower Urinary Tract Symptoms Quality of Life (ICIQ-LUTSqol) outcome measure updated with additional information on Description and Scale<br>o EQ-5D-5L outcome measure updated with additional accurate information on Scale<br>o Addition of "Health Problems" row to outcome measures collected to accurately reflect what is collected at 17 and 52 weeks. Description, Data and Time-points measured column headings filled in<br>o Addition of "additional cancer treatments" row to outcome measures collected at baseline, 17-weeks and 52-weeks. Description, Data and Time-points measured column headings filled in<br>e. Statistical analysis plan updated to state analysis will only be conducted at 52 weeks (end of study) and not at 17 weeks. Clarification also made to state that cost effective analysis will only be completed if clinical effectiveness at 17 weeks is demonstrated. Following sections of protocol changed to reflect these updates:<br>o Protocol Synopsis "Statistical Procedures: Sample Size Calculation & Analysis Plan" (p8)<br>o Section 3.2 Secondary objectives (pp15-16)<br>o Section 6 Study Visits and Procedures Schedule (p33) – box informing analysis at 17 weeks deleted<br>o Section 9.3 Cost Effectiveness Analysis (p39)<br>o Section 12.2 Statistical Analysis plan (pp 41-42)<br>f. Amendment of **Section 11. Adverse Event Reporting** (pp 40-41) to incorporate CTCAE guideline. Grades and attributions updated accordingly. New references relating to CTCAE guidelines added to reference list<br>2. TELE-CONNECT_RCT PICF Master v6 03.06.2024<br>a. Change of title prefix to associate investigator (Lamb) from Dr to A/Prof

		b. Addition of new statistician, Peixuan Li, as Associate Investigator’<br>3. TELE-CONNECT_RCT PICF Community v3 03.06.2024<br>a. Change of title prefix to associate investigator (Lamb) from Dr to A/Prof<br>b. Addition of new statistician, Peixuan Li, as Associate Investigator<br>4. TELE-CONNECT_RCT Baseline Q Booklet_v6 03.06.2024<br>a. Cover pages for Questionnaire (p1) and Accident Diary (p34) updated to include email of second study coordinator, Nipuni Susanto. Missing ACTRN, HREC details added to p34 to match p1.<br>b. In section **C. Cancer specific medical information**, new “additional cancer treatments” question added as Q6 (p8) to collect details on any targeted therapy and/or immunotherapy the participant may have had/ are currently having.<br>5. TELE-CONNECT_RCT 17 week Q Booklet v3 03.06.2024<br>a. Cover pages for Questionnaire (p1) and Accident Diary (p32) updated to include email of second study coordinator, Nipuni Susanto, as well as ACTRN and HREC details.<br>b. In section J**. Additional Questions**, new “additional cancer treatments” question added as Q4 (p31) to collect details on any targeted therapy and/or immunotherapy the participant may have had/ are currently having.<br>c. In section **D. Patient Global Impression of Change (PGIC) & Treatment Satisfaction**, formatting change made to Q2 (p13) where “extremely satisfied” is displayed together under relevant checkbox (previous formatting error rectified)<br>6. TELE-CONNECT_RCT 52 week Q Booklet v3 03.06.2024<br>a. Cover pages for Questionnaire (p1) and Accident Diary (p27) updated to include email of second study coordinator, Nipuni Susanto. Missing ACTRN, HREC details added to p27 to match p1.<br>b. In section **J. Additional Questions**, new “additional cancer treatments” question added as Q4 (p26) to collect details on any targeted therapy and/or immunotherapy the participant may have had/ are currently having.<br>c. In section **D. Patient Global Impression of Change (PGIC) & Treatment Satisfaction**, formatting change made to Q2 (p13) where “extremely satisfied” is displayed together under relevant checkbox (previous formatting error rectified)<br>d. On p33, ‘Unified theory of acceptance and use of technology (UTAUT-II)’ reference deleted from the list of references as this outcome measure is not administered at 52 weeks
01/06/2023	7	- Update selection (inclusion) criterion wording to clarify that patients can participate in the trial via Zoom or any other videoconferencing platform<br>- Update selection (inclusion) criterion related to previous physiotherapy treatments by adding a threshold (no more than 1 treatment) and a time limit (in the previous 4 years) for previous physiotherapy and specifying that sessions could have been held in clinic or via telehealth<br>- Within the selection (inclusion) criteria, removal of upper age limit of 80 years<br>- Update selection (exclusion) criterion related to inability to complete study assessment procedures by adding the wording “including e-health components of study”<br>- Change of last name of associate investigator from Gamage to Susanto<br>- Removal of Western Health as a recruiting site and removal of Dr Richards from the project team list
19/12/2022	6	- Addition of new Associate Investigator, Dr Zobaida Edib<br>- Protocol references corrected<br>- Recruitment process for <u>Private gynaecology clinics and Community (via social media) updated</u><br>- QR code added to community brochure
21/11/2022	5	- Addition of new Associate Investigator, Ms Nipuni Gamage

		- Change eligibility criteria from patients with peritoneal cancer to "primary" peritoneal cancer. As a result, following updated with new wording:<br>o TELE_CONNECT_RCT Baseline Q Booklet_v5 21.11.22<br>o TELE-CONNECT_Eligibility Checklist Health Sites Master v5 21.11.22<br>o TELE-CONNECT_example medical confirmation for community online v2 21.11.22<br>o TELE-CONNECT_Initial online screening Form v4 21.11.22
06/11/2022	4	Minor amendments for HREC<br>1. TELE-CONNECT_RCT Protocol v4 06.11.2022<br>- Change of title prefix to associate investigator (Hyde) from Dr to A/Prof<br>- Within the selection criteria/inclusion criteria addition of patients who have undergone treatment for fallopian tube or peritoneal cancer<br>- Within the selection criteria, age change from over 18 to 18-80 years<br>- Updated selection criteria to specify the patient's adjuvant therapy completed at least 3 months ago<br>- Addition of specific detail of the process regarding recruitment of women from the community. If the woman's medical records are not held at either of the 4 main hospital sites, women will be asked to provide written evidence of their cancer diagnosis and treatment. If they are unable to do this, confirmation will be requested from the woman's GP or specialist.<br>2. TELE-CONNECT_RCT PICF Master v4 25.10.22<br>- Change of title prefix to associate investigator (Hyde) from Dr to A/Prof<br>- Updated inclusion criteria addition of patients who have undergone treatment for fallopian tube and peritoneal cancer<br>3. TELE-CONNECT_RCT PICF Community v1 06.11.22 (new document - created from master v4 06.11.22)<br>- a) Insertion of "community" in location title<br>- b) Removal of the sentence regarding the research team member talking to the women within a clinic setting<br>- c) Addition of specific detail of the process regarding accessing patient's medical notes. If the women's medical records are not held at either of the 4 main hospital sites, women will be asked to provide written evidence of their cancer diagnosis and treatment. If they are unable to do this, confirmation will be requested from the woman's GP or specialist.<br>- d) Addition of the need to receive medical confirmation alongside their consent form<br>- e) Removal of the sentence regarding the need to take part in the research to received treatment at a specific hospital site<br>- f) Addition of all 4 main hospital sites included in the ethics approval letter<br>- g) Addition of "my hospital" instead of specific hospital site<br>4. TELE-CONNECT Eligibility Checklist Health Sites Master v4 06.11.22<br>- a) updated inclusion criteria to addition of patients who have undergone treatment for fallopian tube or peritoneal cancer<br>5. TELE-CONNECT_Initial online screening form v4 06.11.22<br>- a) Updated inclusion criterion addition of patients who have undergone treatment for fallopian tube or peritoneal cancer<br>- b) Addition of word "currently"<br>- c) Addition of question asking if their surgery was at least 6 months ago<br>- d) Addition of question asking to state in which state their treatment occurred<br>- e) Addition of question asking to clarify if chose VIC, which hospital site did they have their cancer treatment<br>- f) Addition of question asking to clarify if chose not VIC, to name which hospital health site they have their cancer treatment<br>- g) Change of numbering from 7 and 8 to 8-12<br>6. TELE-CONNECT_example of medical confirmation for community online v1 06.11.22 (newly created form for community recruitment)

		7. TELE-CONNECT_RCT Baseline Q Booklet_v4 06.11.22<br>- a) on page 7, add locations of fallopian tube or peritoneum to the cancer sites to the available answers
02/05/2022	3	Minor amendments for HREC<br>- a) prior to participant's first physiotherapy consultation, a member of the research team will schedule a Zoom consultation with the participant, in order to practise the technical aspects of internet connection and screen sharing.
29/03/2022	2	Minor amendments for HREC<br>- a) addition of research assistant name<br>- b) change of researcher title<br>- c) updated inclusion criteria to include borderline ovarian tumour (BOT)<br>- d) updated inclusion criteria by specifying the primary cancer treatment<br>- e) addition of specific details of the role of the research assistant in the randomisation process<br>- f) change to the number of consultations the participant will receive in the intervention group, from 8-12 to 8 only<br>- g) change to the home exercise component in the intervention group i.e. participants will not use the biofeedback sensor for home exercise, they will only use it during the consultations with the physiotherapist. When doing their home exercises, they will use the femfit® app on their smartphone or tablet and additional biofeedback strategies taught by the physiotherapist<br>- h) altered name Femfit to femfit®

Current Date: 12/07/2024

Contents

STATEMENT OF COMPLIANCE 1

PROTOCOL SYNOPSIS 7

GLOSSARY OF ABBREVIATIONS 9

1. Study Management 10

1.1 Principal Investigator 10

1.2 Associate Investigators 10

1.3 Statisticians 11

1.4 Internal Trial Monitoring Committee 11

1.5 Advisory Committee meetings 11

1.6 Sponsor 12

1.7 Funding and resources 12

2. INTRODUCTION AND BACKGROUND 12

2.1 Lay Summary 12

2.2 Background Information 12

2.3 Research Question 13

2.4 Rationale for Current Study 13

3. STUDY OBJECTIVES 15

3.1 Primary Objective 15

3.2 Secondary Objectives 15
4. STUDY DESIGN 16
4.1 Type of Study 16
4.2 Study Design 16
4.3 Number of Participants 16
4.4 Study sites 16
4.5 Expected Duration of Study 17
4.6 Primary and Secondary Outcome Measures (follow-up time-points are relative to randomisation) 18
5. PARTICIPANT ENROLLMENT AND RANDOMISATION 25
5.1 Recruitment 25
5.2 Eligibility Criteria 28
5.2.1 Inclusion Criteria 28
5.2.2 Exclusion Criteria 29
5.3 Informed Consent Process 31
5.4 Enrolment and Randomisation Procedures 31
5.5 Blinding Arrangements 32
5.6 Participant Withdrawal 32
5.7 Trial Closure 32
5.8 Continuation of therapy 32
6. STUDY VISITS AND PROCEDURES SCHEDULE 33
7. DATA COLLECTION 34
8. INTERVENTIONS 34
8.1 Usual care (Control) 34
8.2 Telehealth-delivered pelvic floor muscle training program (Intervention) 34
9. ASSOCIATED PROCEDURES/STUDIES 38
9.1 Piloting of the intervention 38
9.2 Qualitative evaluation 39
9.3 Cost effectiveness analysis 39
10. PARTICIPANT SAFETY 40
10.1 Risk Management and Safety 40
11. ADVERSE EVENT REPORTING 40
12. STATISTICAL METHODS 41
12.1 Sample Size Estimation 41
12.2 Statistical Analysis Plan 41

12.3 Interim Analyses 42
13. DATA MANAGEMENT 42
13.1 Data Collection & Storage 42
13.1.1 Identifiable data 42
13.1.2 Re-identifiable/coded data 43
13.2 Data Confidentiality 43
13.3 Study Record Retention 43
14. PROTOCOL DEVIATIONS 44
15. ADMINISTRATIVE ASPECTS 44
15.1 Independent HREC approval 44
15.2 Participant reimbursement 44
15.3 Financial disclosure and conflicts of interest 44
16. USE OF DATA AND PUBLICATIONS POLICY 44
17. REFERENCES 46

PROTOCOL SYNOPSIS

Title	Telehealth exercise for continence after gynaecological cancer treatment (TELE-CONNECT): a randomised controlled trial protocol
Objectives	Primary aim is to investigate if a telehealth-delivered pelvic floor muscle training program incorporating a novel biofeedback device reduces urinary incontinence (primary outcome) compared with usual care, following gynaecological cancer.
Study Design	A 2-group pragmatic superiority randomised controlled trial (RCT)
Planned Sample Size	72 participants
Selection Criteria	Participants will be i) women treated for Stage I, II or III histologically confirmed uterine, endometrial, cervical, fallopian tube, primary peritoneal or ovarian tumour or borderline ovarian tumour; ii) primary cancer treatment completed ≥ 6 months ago or adjuvant therapy completed ≥ 3 months ago; iii) self-reported urinary incontinence (≥ 1 episode per week for the last 4 weeks); iv) have not received >1 pelvic floor physiotherapy treatment for urinary incontinence (in clinic or telehealth) since primary cancer treatment started or in the previous 4 years (whichever is most recent); and v) able to give informed consent and to participate fully in the interventions and assessment procedures (including the e-health components of the study); vi) aged 18 years or older
Study Procedures	Following informed consent and baseline assessment, participants will be randomly allocated to receive either:<br>i) an intensive, supervised, 16-week telehealth-delivered pelvic floor muscle training program including 8 video-consultations with a pelvic floor physiotherapist.<br>ii) usual care (control).<br><br>Participants will be re-assessed via web-based questionnaires at 17 weeks (primary time-point) after commencement of treatment.

	A participant follow-up will be conducted at 52-weeks post randomisation to examine effect maintenance in the same outcomes as 17 weeks, plus a cost effectiveness analysis.
Statistical Procedures: Sample Size Calculation & Analysis Plan	**Sample Size Calculation:** We aim to detect the minimal clinically important difference (MCID) over 17 weeks in the primary outcome of change in urinary incontinence measured using the International Consultation on Incontinence – Urinary Incontinence Short Form (ICIQ-UI SF). The MCID in previous trials is a 2.5 unit change. The sample size calculation accounts for potential clustering by physiotherapists in the intervention arm. Based on previous research, we assume a conservative between-participant standard deviation of 3.2 units. We have also assumed a conservative correlation between baseline and 17-week scores of 0.4, an intra-cluster correlation of 0.05 and 3 physiotherapists treating approximately 10 patients each. With these parameters, we need 30 women per arm to achieve 80% power to detect the MCID at a 0.05 significance level. Allowing for 15% attrition, we will recruit 36 women per arm (in total n=72).<br><br>**Analysis Plan:** A statistical analysis plan will be developed and published prior to unblinding and analysis. All available data from all randomised participants will be used in the analysis. Main comparative analyses between groups will be performed using intention-to-treat. Constrained longitudinal data analysis will be used to analyse continuous outcomes, including the primary outcome. The response will consist of all continuous outcomes (baseline, 17 weeks and 52 weeks) and the model will include factors representing treatment group, time (categorical), and a group-by-time interaction, with the restriction of a common baseline mean across treatment groups. Models will include the stratification variable (radiotherapy) and random effects for physiotherapist (intervention arm only). The mean change in urinary incontinence (primary outcome) and other continuous outcomes from baseline to each follow-up time-point between the two intervention groups will be obtained. The primary hypothesis will be evaluated by obtaining the estimated differences between the two intervention arms in mean change in urinary incontinence score from baseline to 17-weeks post-commencement of treatment (primary time point), two-sided 95% confidence intervals and p-values. These models provide valid inference in the presence of missing data if the data are missing at random. Binary outcomes will each be compared between groups separately using logistic regression, adjusting for the stratifying variable of radiotherapy, and fit using generalized estimating equations to account for clustering, with results reported as risk ratios and risk differences. Poisson regression models fitted using generalized estimating equations will be used for count outcomes.
Duration of the study	Each participant will be involved for 52 weeks.

GLOSSARY OF ABBREVIATIONS

ABBREVIATION	TERM
AE	Adverse Event
ANCOVA	Analysis of Covariance
BMI	Body mass index
BOT	Borderline ovarian tumour
CERT-PFMT	Consensus on Exercise Reporting Template for Pelvic Floor Muscle Training
CI	Confidence Interval
CONSORT	Consolidated Standards of Reporting Trials
DOB	Date of birth
HEP	Home exercise program
ICIQ-UI SF	International Consultation on Incontinence Questionnaire: Urinary Incontinence – Short Form
ICIQ-LUTSqol	International Consultation on Incontinence Questionnaire Lower Urinary Tract Symptoms Quality of Life
MCID	Minimal clinically important difference
NHMRC	National Statement on Ethical Conduct in Human Research
NRS	Numeric Rating Scale
PFBQ	Pelvic Floor Bother Questionnaire
PFM	Pelvic floor muscle
PFMT	Pelvic floor muscle training
PGIC	Patient Global Impression of Change
PICF	Participant Information and Consent Form
RCT	Randomized Controlled Trial
SD	Standard Deviation
SPIRIT	Standard Protocol Items: Recommendations for Interventional Trials
TGA	Therapeutic Goods Administration
UTAUT-II	Unified theory of acceptance and use of technology
VCA	Victorian Cancer Agency

1. Study Management

1.1 Principal Investigator

Name	Role	Contact information
A/Prof Helena Frawley	A/Prof Frawley is a women's health physiotherapy clinical researcher, specialising in pelvic floor function and dysfunction. She will lead the development, conduct, reporting, dissemination and translation of the RCT and will be responsible for all ethical and governance aspects of this project. She will also be responsible for publishing the trial protocol and the findings of the trial.	P: +61 0418 584 813<br>E: h.frawley@unimelb.edu.au<br><br>Faculty of Medicine, Dentistry & Health Sciences<br>School of Health Sciences<br>University of Melbourne VIC 3010

1.2 Associate Investigators

Name	Role	Contact information
Prof Linda Denehy	Professor Denehy is a PhD-qualified physiotherapist who is Head of the Melbourne School of Health Sciences at the University of Melbourne. She will provide expertise in conducting the trial and reporting of the findings.	P: +61 3 8344 6428<br>E: l.denehy@unimelb.edu.au<br><br>Faculty of Medicine, Dentistry & Health Sciences<br>School of Health Sciences<br>University of Melbourne VIC 3010
Prof Kim Bennell	Prof Bennell is a research physiotherapist and Director of the Centre for Health, Exercise and Sports Medicine (CHESM). She will provide expertise in conducting the trial and reporting of the findings.	P: 03 8344 4135<br>E: k.bennell@unimelb.edu.au<br><br>Centre for Health, Exercise and Sports Medicine<br>Department of Physiotherapy<br>Faculty of Medicine, Dentistry & Health Sciences<br>School of Health Sciences<br>University of Melbourne VIC 3010
Dr Rachel Nelligan	Dr Nelligan is a physiotherapist and clinical researcher. She has experience in the set-up and coordination of RCTs involving digital health interventions, as well as qualitative methods. She will assist with co-ordination of the trial.	P: N/A<br>E: Rachel.nelligan@unimelb.edu.au<br><br>Department of Physiotherapy<br>School of Health Sciences<br>University of Melbourne VIC 3010
Angela Ravi	Mrs Ravi is a physiotherapist at Mercy Hospital for Women and research assistant for this trial.	P: 0434772748<br>E: angela.ravi@unimelb.edu.au<br><br>Department of Physiotherapy<br>School of Health Sciences<br>Faculty of Medicine Dentistry & Health Sciences<br>University of Melbourne VIC 3010
A/Prof Orla McNally	A/Prof McNally is the Director Oncology/Dysplasia at the Royal Women's Hospital. She will play a supportive role in recruitment to this study.	P: 03 83453562<br>E: orla.mcnally@thewomens.org.au<br><br>Royal Women's Hospital<br>Parkville VIC 3052
A/Prof Simon Hyde	A/Prof Hyde is the Director of Gynaecological Oncology at Mercy Hospital for Women. He will	P: 03 84584860<br>E: SHyde@mercy.com.au

	play a supportive role in recruitment to this study.	Mercy Hospital for Women, Heidelberg, VIC 3084
Dr Shih-Ern Yao	Dr Yao is a consultant of Gynaecologic Oncology at Monash Medical Centre, Melbourne. He will be the lead advisor in gynaecologic oncology aspects of the project, and will play a supportive role in recruitment to this study.	P: 03 9928 8243<br>E: shih-ern.yao@monashhealth.org<br><br>Department of Gynaecologic Oncology Monash Health, Melbourne VIC 3168
Ms Nipuni Susanto	Ms Susanto is a Research Assistant for this trial with experience in coordinating investigator initiated studies and clinical trials.	P: 0432 855 531<br>E: nipuni.susanto@unimelb.edu.au<br><br>Faculty of Medicine, Dentistry & Health Sciences<br>School of Health Sciences<br>University of Melbourne VIC 3010
Dr Zobaida Edib	Dr Edib is a Research Assistant with experience in coordinating investigator-initiated studies. She will be assisting with recruitment and other research related activities for this trial.	P: 0470630182<br>E: zobaida.edib@unimelb.edu.au<br><br>Faculty of Medicine, Dentistry & Health Sciences<br>School of Health Sciences<br>University of Melbourne VIC 3010

1.3 Statisticians

A/Prof Karen Lamb	P: 03 8344 8701<br>E: klamb@unimelb.edu.au<br><br>Centre for Epidemiology and Biostatistics<br>Melbourne School of Population and Global Health<br>University of Melbourne VIC 3010
Fiona McManus	P: 03 8344 8701<br>E: fmcmanus@unimelb.edu.au<br><br>Centre for Epidemiology and Biostatistics<br>Melbourne School of Population and Global Health<br>University of Melbourne VIC 3010
Peixuan Li	P: 03 8344 8701<br>E: li.p4@unimelb.edu.au<br><br>Centre for Epidemiology and Biostatistics<br>Melbourne School of Population and Global Health<br>University of Melbourne VIC 3010

1.4 Internal Trial Monitoring Committee

The Principal Investigator, the Trial Coordinator, and other invited Investigator(s) will meet fortnightly to monitor progress of the trial, including any reported adverse events.

1.5 Advisory Committee meetings

Two meetings each year will be conducted with the Advisory Committee to oversee trial progress and conduct. The Advisory Committee will be made up of the Principal Investigator, members of the research team and 3 consumer members.

1.6 Sponsor

The University of Melbourne.

1.7 Funding and resources

Funding has been provided by the Victorian Cancer Agency (Grant ID: MCRF20029).

2. INTRODUCTION AND BACKGROUND

2.1 Lay Summary

This study (called TELE-CONNECT) is a telehealth program to deliver pelvic floor muscle training to women with gynaecological cancer who suffer from urinary incontinence. Urinary incontinence affects around one-third of women causing significant physical, social, emotional and financial burden. The prevalence of incontinence is doubled in women with gynaecological cancer. Although evidence supports pelvic floor muscle training as first-line treatment for urinary incontinence, it is not known whether this treatment is as effective for women following gynaecological cancer treatment. This will be the first randomised trial to address this vital clinical question using the novel method of telehealth.

2.2 Background Information

Urinary incontinence is the most prevalent type of pelvic floor disorder, affecting 31% of Australian women[1]. A recent systematic review of pelvic floor disorders following treatment for gynaecological cancer reported that the prevalence of urinary incontinence was 76% following cervical cancer and 84% following uterine cancer[2].

Incontinence impacts on a woman's social and emotional wellbeing and ability to engage with the community, with 39% of women who experience incontinence reporting that they are less confident in leaving the house, 32% suggesting that it affects their mental health and wellbeing and 25% indicating that it affects their relationships with family and friends[1]. The majority of women do not disclose incontinence nor seek help, due to stigma, the intimate nature of their symptoms and the perception of incontinence as being normal[1], yet incontinence significantly diminishes a woman's quality of life[3]. Pelvic floor disorders have been called a 'hidden epidemic'[4]. This silence also affects women with gynaecological cancer, who express resignation of their incontinence as a consequence of being cancer-free[5, 6].

Gynaecological cancer is the third most commonly diagnosed cancer in Australian women, with around 6,652 newly diagnosed women in 2020[7]. With increasing survival rates, quality of life in this population is becoming more important. Because the prevalence and impact of incontinence is higher in gynaecological cancer survivors compared to the general population[8], this burden is likely to negatively impact survivor's ability to re-engage with life post-cancer treatments.

Pelvic floor muscle training is a low-risk, low-cost exercise therapy with Level 1A evidence for women with urinary incontinence[9]. Supervised, intensive pelvic floor muscle training changes muscle physiology and morphometry[10]. Women with stress urinary incontinence (the most prevalent type of incontinence) who undertook pelvic floor muscle training were six times more likely to report cure or improvement (74% versus 11%; RR 6.33, 95% CI 3.88 to 10.33) than controls, and women with any kind of urinary incontinence who perform pelvic floor muscle training are twice as likely to report cure or improvement than control groups (67% versus 29%; RR 2.39, 95% CI 1.64 to 3.47)[9].

Due to potential effects of gynaecological cancer and its treatment (surgery, radiotherapy and chemotherapy) on pelvic floor structures [11-13], gynaecological cancer survivors may respond differently to pelvic floor muscle training compared to non-cancer populations and may require more intensive or longer duration training in order to achieve the same improvement as the non-cancer population, therefore population-specific investigation is required. Only two small trials have investigated pelvic floor muscle training to treat urinary incontinence in women following gynaecological cancer with mixed results [14, 15]. Our systematic review of gynaecology cancer trials highlighted the need for further studies to test pelvic floor muscle training, using effective exercise dosage, adherence, supervision and progression to measure efficacy in gynaecological cancer survivors [16]. Improving quality of life following gynaecological cancer treatments is an identified priority [17]. As urinary incontinence is the most prevalent pelvic floor disorder following treatment for gynaecological cancer and has such a distressing impact on quality of life in survivors[6, 8, 18], this condition in this population is an urgent priority for research. Effective treatments that provide equity of access in a format that is acceptable to women with urinary incontinence following gynaecological cancer are required.

We anticipate that traditional face-to-face clinical trials will be challenging to conduct in the near future, particularly in vulnerable populations. Even when COVID-19 restrictions lift, clinicians will need to provide evidence-based treatment for women with urinary incontinence following gynaecological cancer to women remote from specialised services or those who prefer the convenience of video-consultations. Telehealth may prove an effective option in this circumstance. Due to the recent restrictions (SARS-COV2), clinicians have rapidly embraced this method of healthcare delivery. Our interim data from a current survey of pelvic floor physiotherapists and patients who have converted from in-person to video-consultations for incontinence treatment show high levels of patient-perceived effectiveness, satisfaction with the service, and value placed on the convenience, reduced waiting time, privacy and access to this model of service delivery (Bennell, unpublished data).

This model of care delivery will also provide an option for those close to services, who prefer home-based care. If a telehealth-delivered pelvic floor muscle training intervention effectively improves urinary incontinence this represents a potential new approach to cost-effective, evidence-based care which will increase access for rural and remote women and provide disadvantaged women with equity of care.

2.3 Research Question

Does a 16-week, physiotherapist-supervised, telehealth-delivered pelvic floor muscle training program lead to significantly greater and clinically meaningful reductions in urinary incontinence, when compared to a usual care control at 16 weeks?

2.4 Rationale for Current Study

Why is managing urinary incontinence following gynaecological cancer important?

The National Framework for Gynaecological Cancer Control recommends research to assess effectiveness of models of follow-up and survivorship[17]. Despite the high prevalence, severity and distress of urinary incontinence in gynaecological cancers[2, 8, 18], screening for symptoms of urinary incontinence is not routine and is not addressed in current Cancer Council care pathways. This represents an unmet patient need.

Why does urinary incontinence occur following gynaecological cancer and could pelvic floor muscle training work?
While the overall aim of medical and surgical treatments of gynaecological cancer is curative, the treatments themselves may contribute to the morbidity of pelvic floor disorders through direct neuro-muscular and vascular damage to pelvic structures [11-13] as well as impact of reduced oestrogen on pelvic tissues through surgically-induced menopause [19, 20]. These cancer-treatment sequalae are likely causes of the increased prevalence of pelvic floor disorders in this population.

Despite these cancer treatments' impacts on the pelvic floor, two pilot randomised controlled trials (RCTs) have shown improved pelvic floor muscle strength following pelvic floor exercise in women after gynaecological cancer treatment, confirming the muscle can respond to an exercise stimulus[14, 15]. The earlier study found no improvement in urinary incontinence, as the study was not powered to find a difference in this outcome[14]. The second study reported a Patient Global Impression of Improvement in incontinence after one supervised pelvic floor muscle training instruction session and 12 weeks of home exercise, but no improvement in condition-specific quality of life or bother from incontinence[15], possibly due to insufficient exercise supervision. Further evidence to establish if dose-effective, supervised pelvic floor muscle training can improve frequency, severity and bother of urinary incontinence in women following treatment for gynaecological cancer is urgently required.

What are barriers to pelvic floor muscle training for women following gynaecological cancer in the current model of care?
Qualitative studies reveal gynaecological cancer survivors receive little or no information about treatments for potential pelvic floor pelvic floor symptoms[5] yet would be willing to try pelvic floor muscle training if given the option[6]. Our own research from interviewing patients and clinicians highlights barriers to provision and uptake of pelvic floor muscle training: lack of access to treatment, priority of cancer over other health issues, acceptance of the sequelae of cancer treatments, psychological effects of gynaecological cancer on a woman's sexual self-identity and emotional and physical trauma of undergoing multiple vaginal examinations [21]. Treatments need to be provided in novel ways to overcome existing challenges and facilitate patient uptake, to give women with urinary incontinence following gynaecological cancer the same opportunities to access care as others in the community, in a format that is acceptable to them.

A novel and timely strategy to deliver pelvic floor muscle training to treat urinary incontinence
Telehealth may provide a means to overcome service and access barriers, reduce appointment fatigue, and be an opportunity not just for rural and remote gynaecological cancer patients, but also those who prefer home-based care. Evidence for telehealth-delivered pelvic floor muscle training to treat urinary incontinence has been synthesised in a recent systematic review, and a strong effect was reported: a significant reduction in urinary incontinence severity (SMD = −0.90, 95% CI, −1.73 to −0.07) and improved quality of life (SMD= 0.71, 95% CI, 0.21 to 1.20) [22]. However, none of the seven studies compared pelvic floor muscle training delivered by a pelvic floor physiotherapist via video-consultations with usual care, and none included women with gynaecological cancer. This delivery method is promising and potentially overcomes many known system-level and patient barriers to continence care[23].

Can pelvic floor muscle training be delivered via telehealth?
A unique challenge of telehealth-delivered pelvic floor muscle exercise intervention is the inability of the clinician or patient to visualise the pelvic muscle contraction. It is essential to know that telehealth instruction results in the correct muscle action, the foundation of effective pelvic floor muscle training.

Written or verbal instructions alone are insufficient for up to 50% of women[24], therefore standard clinical practice is to undertake a vaginal examination to verify correct pelvic muscle contraction and facilitate a pelvic floor muscle response[25]. Recently, a new and innovative intra-vaginal pressure biofeedback device (femfit®) has been developed to confirm correct pelvic floor muscle contraction and reliability and validity of this device has been demonstrated[26, 27]. We will include this novel device to facilitate remote teaching of a correct pelvic floor muscle contraction[28].

We will test the effectiveness of the first telehealth-delivered pelvic floor muscle training program that combines detailed verbal instruction and supervision from a pelvic floor physiotherapist with patient home-use of the biofeedback device, via video-consultation. Our program will include the essential elements of effective pelvic floor muscle training programs: confirmation of correct contraction, intensive supervision by a physiotherapist and regular training[28, 29]. Our systematic review of physical activity in women following gynaecological cancer demonstrated low levels of physical activity[30], which may reflect poor exercise behaviour generally. We will incorporate evidence-based strategies to maximise uptake and adherence to muscle training identified in our previous research[31-33].

3. STUDY OBJECTIVES

3.1 Primary Objective

The primary aim of this pragmatic randomised controlled trial (RCT) is to investigate if a telehealth-delivered pelvic floor muscle training program incorporating a novel biofeedback device, reduces urinary incontinence (primary outcome) compared with usual care, in women following treatment for a histologically confirmed uterine, endometrial, cervical, fallopian tube, primary peritoneal or ovarian tumour (Stage I, II or III) or borderline ovarian tumour (BOT), a tumour which has micro-invasion into surrounding tissues and scheduled for hysterectomy and bilateral salpingo-oopherectomy, at 17 weeks.

The primary hypothesis is that there will be a reduction in urinary incontinence at 17 weeks, measured on the International Consultation on Incontinence Questionnaire: Urinary Incontinence – Short Form (ICIQ-UI SF)[34] (primary outcome measure), in favour of the intervention arm.

3.2 Secondary Objectives

1. Aim: to investigate differences in quality of life (QoL) and condition-specific health-related QoL, bother of pelvic floor symptoms, leakage episodes, use of continence pads, global impression of change and the proportion that met or exceeded MCIDs for the primary outcome [35] (secondary outcomes) between the telehealth-delivered pelvic floor muscle training program incorporating a novel biofeedback device and usual case.

2. Aim: to evaluate processes within this trial that, if found to be effective, will assist future translation of the findings into clinical practice, relating to fidelity, reach, effectiveness, adoption, implementation and maintenance of the intervention.

3. Mechanistic aim: to investigate if the intervention improves pelvic floor muscle strength in women in the intervention arm, at 17-weeks.

4. Aim: To assess differences in the primary and secondary outcomes at 52-weeks (secondary time point) to assess any maintenance in treatment effect.

5. Aim: To assess cost effectiveness at 52-weeks if clinical effectiveness at 17-weeks is demonstrated.

6. Aim: To conduct an exploratory analysis of whether the effects of the intervention are moderated by pre-specified moderators (radiotherapy: categorical variable [yes(type)/no]; BMI: continuous variable).

4. STUDY DESIGN

4.1 Type of Study

A co-designed pragmatic superiority RCT.

4.2 Study Design

This is a pragmatic, 2-group stratified, assessor-blinded, block-randomized clinical trial, to focus on the effectiveness outcomes of the intervention while also exploring the implementability of the intervention (type 1 hybrid trial) [36] and complies with SPIRIT guidelines [37]. Reporting will comply with CONSORT and appropriate extensions [38-40] and the Consensus on Exercise Reporting Template for Pelvic Floor Muscle Training (CERT-PFMT) guidelines[41].

Seventy-two women will be recruited from public outpatient clinics of participating health services and the community. Additionally, if needed to meet recruitment targets, women may also be recruited via the private outpatient rooms of already participating consultants. If this is required, an ethics amendment will be submitted and recruitment in private clinics will only commence after the amendment has been approved. Participants are women following cancer treatment (with and without radiotherapy) for Stage I, II or III uterine, cervical, fallopian tube, primary peritoneal or ovarian cancer or BOT with primary/planned treatment completed ≥ 6 months ago or adjuvant therapy completed ≥ 3 months ago. Multi-site ethics approval will be obtained from the Human Research Ethics Committee of Monash Health.

4.3 Number of Participants

We will recruit 36 participants per treatment arm; therefore 72 participants will be recruited in total, or more, as required to meet the minimum number of women per arm (30 women per arm to achieve 80% power to detect the MCID at a 0.05 significance level), depending on actual attrition between baseline and primary follow-up time-point

4.4 Study sites

Site	Address	Contact Person	Phone	Email
The Royal Women's Hospital	Grattan St and Flemington Rd, Parkville, VIC 3052	A/Prof Orla McNally Director Oncology/Dysplasia	03 83453562	Orla.McNally@thewomens.org.au
Mercy Hospital for Women	163 Studley Rd, Heidelberg, VIC 3084	A/Prof Simon Hyde Director of Gynaecological Oncology	03 84584860	SHyde@mercy.com.au

Monash Health	823-865 Centre Rd, Bentleigh East, VIC 3165	Dr Shih-Ern Yao Consultant of Gynaecological Oncology	03 9928 8243	shih-ern.yao@monashhealth.org

4.5 Expected Duration of Study

Anticipated participant recruitment commences: April 2022. The anticipated and conservative rate of recruitment is approximately 10 women per quarter (April 2022 – Dec 2023). With the last patient recruited in December 2023, and their 17-week follow-up completed by May 2024, we anticipate data collection will be completed by June 2025.

4.6 Primary and Secondary Outcome Measures (follow-up time-points are relative to randomisation)

Measure name	Description	Scale	Time-points measured	Statistical analysis plan
**Primary Outcomes**				
International Consultation on Incontinence Questionnaire: Urinary Incontinence – Short Form (ICIQ-UI SF) [34]	A 3-item self-reported questionnaire capturing frequency, severity and bother of any type of urinary incontinence in the past 4 weeks. Scored by summing the responses to 3-items. Items are summed to create one overall score.	Ranges from 0-21.<br>Higher scores indicate greater impact of UI.	Baseline, 17 and 52 weeks	Constrained longitudinal data analysis across all time points, accounting for stratification factor and physiotherapist.
**Secondary Outcomes**				
International Consultation on Incontinence Questionnaire Lower Urinary Tract Symptoms Quality of Life (ICIQ-LUTSqol) [42]	A 20-item self-reported questionnaire capturing the impact of any type of urinary incontinence on quality of life.<br><br>Items are summed to create one overall score.<br><br>Domain-specific scores within seven domains (role limitation, physical limitations, personal relationships, emotions, sleep/energy, severity measures) will also be calculated.	Overall score ranges from 19-76.<br>Higher scores indicating increased impact on quality of life.<br><br>The domain-specific scores will be calculated using a specific scoring method developed by the ICIQ group. Domain scores range from 0 (best) to 100 (worse)	Baseline, 17 and 52 weeks	Constrained longitudinal data analysis across all time points, accounting for stratification factor and physiotherapist.
Pelvic Floor Bother Questionnaire (PFBQ) [43]	A 9-item self-reported questionnaire capturing bother of pelvic floor symptoms including stress urinary incontinence, urinary urgency and frequency, urge incontinence, dysuria, pelvic organ prolapse, obstructed defecation, faecal incontinence, and dyspareunia.<br><br>Each item is scored in a range from 0 to 5 with higher scores indicating more severe bother.	Ranges from 0 to 45.<br><br>To have a summary score ranging from 0 to 100, the total score can be transformed by multiplying the mean score of the answered items by 20.	Baseline, 17 and 52 weeks	Constrained longitudinal data analysis across all time points, accounting for stratification factor and physiotherapist.

EQ-5D-5L [44, 45]	Health-related quality of life is self-reported via<br>- five dimensions: mobility, self-care, usual activities, pain/discomfort and anxiety/depression. Each dimension has 5 levels: no problems, slight problems, moderate problems, severe problems and extreme problems (descriptive component) and<br>- one additional self-rated health question scored on a visual analogue scale ranging from “the best health you can imagine” to “the worst health you can imagine” (evaluation component).	Ranges from -0.285 to 1.00; a negative value represents a health state that is worse than death.<br><br>An index score will be calculated as 1 minus the total utility captured in the EQ-5D-5L<br><br>The visual analogue scale score ranges from 0 -100	Baseline, 17 and 52 weeks	Constrained longitudinal data analysis across all time points, accounting for stratification factor and physiotherapist.
Unified theory of acceptance<br>and use of technology (UTAUT-II) [46, 47]	A 26-item self-reported custom-built questionnaire modified from the UTAUT-II model.<br><br>Items are divided within 9 domains:<br>1. Performance expectancy (3-items)<br>2. Effort expectancy (4-items)<br>3. Social influences (3-items)<br>4. Facilitating conditions (4-items)<br>5. Hedonic motivation (3-items)<br>6. Price value (3-items)<br>7. Habit (3-items)<br>8. Behavioural intention (3-items)<br>9. Use behaviour (3-items)	For 3-item domains: ranges from 3-21.<br><br>For 4-item domains: ranges from 4-28.<br><br>Higher scores indicate greater acceptance/use.	Baseline and 17 weeks	Constrained longitudinal data analysis across both time points, accounting for stratification factor and physiotherapist.

	Each item is scored on a 7-point Likert scale ranging from 1-7.<br><br>Items are summed to create one score for each domain.			
Number and severity of leakage episodes	Collected by the participant in a custom-developed 7-day Accident Diary.	Whole numbers/ counts from 0 overall and within categories of damp, wet and soaked. Reported as mean (SD) or median (IQR).	Baseline, 17 and 52 weeks	Poisson regression using generalised estimating equations to account for clustering, adjusting for stratification factor and physiotherapist.
Number of leakage episodes by provocation	Collected by the participant in a custom-developed 7-day Accident Diary.	Whole numbers/counts from 0 within seven categories of provocation: i) urgency urinary incontinence, ii) stress urinary incontinence, iii) mixed urinary incontinence, iv) post-micturition leakage, v) nocturnal enuresis, vi) postural urinary incontinence, vii) unknown	Baseline, 17 and 52 weeks	Poisson regression using generalised estimating equations to account for clustering, adjusting for stratification factor and physiotherapist.
Number of continence pads used	Collected by the participant in a custom-developed 7-day Accident Diary.	Whole numbers/ counts from 0. Reported as mean (SD) or median (IQR).	Baseline, 17 and 52 weeks	Poisson regression using generalised estimating equations to account for clustering, adjusting for stratification factor and physiotherapist.
Patient Global Impression of Change (PGIC) Global Rating of Change Scale	Self-reported in response to “Compared to your first assessment in this study (when you completed your baseline information 17 weeks ago) overall, how would you rate your bladder control now?	Participants indicating they are “much improved” or “very much improved” will be classified as improved. All other respondents will be classified as not improved.	17 and 52 weeks	Logistic regression using generalised estimating equations to account for clustering, adjusting for stratification factor and physiotherapist.

	Responses will be captured on a on a 7-point Likert scale ranging from 1=very much worse to 7= very much improved			
International Physical Activity Questionnaire Short last 7 days Self-Administered Format [48]	A 4-item self-reported questionnaire capturing time spent (days, hours, minutes) on vigorous physical activities, moderate activities, walking and sitting in the past 7 days.<br><br>Responses can be converted into categorical and/or continuous scores.<br><br>Categorical: converted into two physical activity categories<br>Category 1: Low / inactive<br>Category 2: Moderate or High<br><br>Continuous: reported as median METminutes for walking (W), moderate-intensity activities (M), and vigorous-intensity activities (V)<br>(METs are multiples of the resting metabolic rate).	Categorical: reported as number and %<br><br>Or<br><br>Continuous: counts from 0 reported as median values and interquartile ranges.	Baseline, 17 and 52 weeks	Constrained longitudinal data analysis across all time points (continuous outcome), accounting for stratification factor and physiotherapist.<br><br>Logistic regression (binary outcome) using generalised estimating equations to account for clustering, adjusting for stratification factor and physiotherapist.

**Baseline descriptive measures**	Description	Data	Time-point measured
Date of Birth (DOB)	DOB will be patient reported and used to calculate age by subtracting from the date at baseline questionnaire completion.	DOB: DD/MM/YYYY<br>Age: years	Baseline
Height	Self-reported<br>"What is your height?"	Measured in cm	Baseline
Weight	Self-reported<br>"How much do you weigh?"	Measured in kg	Baseline
Body mass index (BMI)	Calculated from self-reported height and weight	Reported in kg/m^2	Baseline
Postcode	Self-reported<br>"What is your postcode?"	Reported as metropolitan/regional derived from the Australian Bureau of Statistics remoteness index. Postcode will also be used to present socioeconomic status	Baseline

		according to Australian Bureau of Statistic guidelines.	
Current employment status	Self- reported in response to “Which of the following best describes your current employment status?”<br>Options:<br>1=Working full time<br>2=Working part time or casual<br>3=Sick leave<br>4=Not employed<br>5=Retired<br>6=Other: (open text)	Reported as a number and %	Baseline
Current relationship status	Self-reported in response to “Which of the following best describes your relationship status?”<br>Options:<br>1=Single<br>2=In a relationship/married<br>3=Other: (open text)	Reported as a number and %	Baseline

**Baseline medical history**	Description	Data	Time-point measured
Birth History	Self-reported in response to:<br><br>1. How many times have you given birth? 0 – 1, 2,3<br>2. How many of your births were vaginal delivery?	Reported as a number and %<br>Mean & SD	Baseline
Hormone status	Hormone status post cancer treatment will be self-reported by asking the participant to select from the list:<br>1=Pre-menopausal<br>2=Peri-menopausal<br>3=Post-menopausal<br>4=Surgical menopause<br>5= Don’t know	Reported as a number and %	Baseline
Pelvic floor problems	Participants will be asked to indicate if they experienced bladder or bowel incontinence prior to cancer treatment:<br>Options:<br>1. Urinary Incontinence<br>2. Faecal Incontinence	Reported as a number and %	Baseline
Co-morbidity	Reported using the Self-Administered Comorbidity Questionnaire [49]	Reported as number and %<br>OR<br>comorbidities per participant will be summed and comorbidities for the cohort will be reported as Mean (SD).	Baseline
**Cancer specific medical history**			
Stage of cancer when diagnosed	Self-reported by selecting from:<br>1. Stage I<br>2. Stage IIA<br>3. Stage IIB<br>4. Stage IIC<br>5. Stage II unsure which level<br>6. Stage IIIA<br>7. Stage IIIB	Reported as number and %	Baseline

	8. Stage IIIC<br>9. Stage III unsure which level<br>10. Recurrent cancer		
Cancer location	Self-reported by selecting from:<br>1. Uterus<br>2. Cervix<br>3. Ovary<br>4. Fallopian tube<br>5. Primary peritoneal	Reported as number and %	Baseline
Time since last treatment for cancer	Self-reported in response to "What date did your last cancer treatment end?" Response converted into months.	Reported as mean (SD) or median (IQR).	Baseline
Past cancer treatment details	Self-reported by selecting from:<br>1. Surgery<br>2. Pre-operative chemotherapy<br>3. Pre-operative radiotherapy<br>4. Post-operative chemotherapy<br>5. Post-operative radiotherapy<br>6. other<br><br>If women had radiotherapy they will be asked if they had<br>i. External beam radiotherapy<br>ii. Brachytherapy	Reported as number and %	Baseline
Vaginal dilator therapy	Participants will be asked if they have done vaginal dilator therapy:<br>1=Yes, I am currently doing<br>2=Yes, but I have stopped<br>3=No, I have not done this	Reported as number and %	Baseline

**Process and other measures**	**Description**	**Data**	**Time-points measured**
Clinical measures	Data relating to PFMT and PFM function will be collected by the femfit® biofeedback device. This will include the magnitude, duration and timing of pressure changes, reflecting the muscle contraction strength, endurance and repetitions.<br>This data will automatically be uploaded and stored on a secure server via the femfit® smartphone app. Data will be exported by the treating study physiotherapist and provided to the research team.	Pressure manometry will be reported in mmHg and seconds	At first and last treatment session
Exercise adherence	Adherence to the prescribed home exercise program will be collected within the femfit® app exercise diary.	Reported as a number and %	17 weeks (intervention group only)
Attendance at physiotherapy video-consultations	The number of physiotherapy consultations completed by each participant will be recorded by the physiotherapist in the treatment notes.	Reported as a number and % of the total scheduled sessions	17 weeks (intervention group only)
Treatment satisfaction	Self-reported in response to "How satisfied are you with the intervention that you received in this study?"<br><br>Responses will be captured on a on a 7-point Likert scale ranging from	Reported as a number and %..	17 and 52 weeks

	1=extremely unsatisfied to 7=extremely satisfied		
Health problems	Change in health status will be defined as any change from the participant's baseline (pre-treatment) condition, other than improvement. Information will be collected within the follow-up questionnaires via a custom-developed table.<br>All changes to health status data will be aggregated.	Types/categories of health changes will be reported. Reported as number and %	17 and 52 weeks
Cancer treatment since commencing this study	Participants will be asked if they have had any cancer treatment since completing their last study questionnaire. If yes, participants will be asked to select which apply<br>1. Chemotherapy<br>2. Radiotherapy<br>3. Surgery<br>4. Other (open text)<br>5. No, I have not had any	Reported as number and %	17, 52 weeks
Additional cancer treatment	Participants will be asked if they have had any targeted therapy or immunotherapy in addition to chemotherapy, radiotherapy and surgery. If yes, participants will be asked to indicate whether they are currently on this/these treatment(s) or to provide stop date.	Reported as a number and %	Baseline, 17, 52 weeks
Medications relevant to pelvic floor function	Self-reported via a custom-developed table listing common medications which can affect bladder, bowel, sexual function. Participants will be asked to select, from the list, which medications they have taken in the past week.	Whole numbers/ counts from 0.<br>Reported as a number and %	Baseline, 17, 52 weeks
Current exercise levels: pelvic floor muscle	Participants will be asked if they currently do pelvic floor muscle exercises and<br>to select how often from options<br>0= Not currently doing<br>1= Less than 1 time a month<br>2= At least 1 time a month<br>3=At least 1 time a week<br>4= At least 3 times a week<br>5= At least 3 times a day	Reported as number and %	Baseline, 17, 52 weeks
Adverse Events	Adverse events (AE) will be defined as any change from the participant's baseline (pre-treatment) condition, other than improvement. Information about AEs will be collected in two ways:<br>i) self-reported within the follow-up questionnaires via a custom-developed table and/or<br>ii) reported directly to the research team as per section 7. 'Adverse event reporting' within this protocol.<br>All AE data will be aggregated.	Type of adverse events and proportions of participants experiencing each adverse event will be reported.	17 and 52 weeks

Health Economic Data	Description	Data	Time-points measured
Cost of physiotherapist's time	The cost of physiotherapy services provided to each participant will be calculated by the research team.	Reported as the dollar cost per participant (mean and SD)	17 weeks (Intervention group only)
Cost of resources	The cost of intervention resources provided to each intervention group participant will be calculated by the research team. Resources include the biofeedback devices and package of participant resources	Reported as the dollar cost per participant (mean and SD)	17 weeks (Intervention group only)
Cost of pads for bladder leakage	To estimate costs of pad use for incontinence we will collect details of<br>- disposable pads including brand name, cost and average number used via Table 1 of the Dowell Bryant Incontinence Cost Patients Index (DBICI)[50].<br>- reusable incontinence underwear including brand and cost.<br>This information will be collected from participants using retrospective recall over the past month.	Reported as the dollar cost per participant (mean and SD)	Baseline, 17 and 52 weeks
Laundry associated with incontinence	Collected from participants using a modified version of Table 3 of the DBICI [50].<br>This information will be used to calculate laundry costs related to incontinence.	Reported as the dollar cost per participant (mean and SD)	Baseline, 17 and 52 weeks
Health service use related to urinary incontinence	Health professional appointments, surgeries, investigations, and medications relating to urinary incontinence will be collected from participants using Section 2 of the DBICI [50].<br>This information will be used to calculate treatment expenditure.	Reported as the dollar cost per participant (mean and SD)	Baseline, 17 and 52 weeks
Work productivity	Information about paid employment or self-employed, leave and job performance will be collected from participants via a custom-built questionnaire	Reported as mean (SD) and number and % where appropriate	Baseline, 17 and 52 weeks

5. PARTICIPANT ENROLLMENT AND RANDOMISATION

5.1 Recruitment

Seventy-two women will be recruited via targeted invitations to eligible women from public outpatient gynae-oncology clinics of participating health services (n=4). We will also recruit women from the community by inviting patients on existing research databases who have consented to be involved in future research to participate, and via advertisements through community foundations/social media/care group advertisements (e.g. Counterpart, Australian Cervical Cancer Foundation, Ovarian Cancer Foundation, twitter). Study advertisements to recruit women from the community will include a link to an online form that contains detailed study information (information taken from the PICF) and a brief online survey where women can assess their initial eligibility for the study. Women who complete the online survey and are potentially eligible will then receive a follow up call from the research team to discuss the study in greater detail and confirm full eligibility (see 'Example Online/Social Media Advertisement' and 'Initial Online Screening Form' documents). Additionally, if needed to meet recruitment targets, women may also be recruited via the private outpatient rooms of already

participating consultants. If this is required, an ethics amendment will be submitted and recruitment in private clinics will only commence after the amendment has been approved.

Potentially eligible women (those receiving treatment for histologically confirmed uterine, endometrial, cervical, fallopian tube, primary peritoneal or ovarian tumour or BOT, International Federation of Gynecology and Obstetrics (FIGO) cancer staging system classification stages I-III) will be identified and approached to participate by staff working at the relevant health services, or will respond to flyers/advertisements (see example in 'Recruitment Brochure'). Women identified as potentially eligible by clinical staff (using the 'Eligibility Checklist Health Sites' form), and who agree to be contacted by a member of the research team, will be contacted by a research team member via phone who will provide detailed information about the study and undertake further screening to confirm eligibility (using the 'Eligibility Screening Form' form). Details of the recruitment processes at each health site are outlined below and summarised in Figure 1. Potentially eligible women will have as much time as they desire to consider participation, within the recruitment timeframe of the study (anticipated participant recruitment is April 2022 - December 2023).

We anticipate 80 women per quarter (from all sites and sources) will be eligible on cancer type and treatment criteria; 50% of these will have urinary incontinence (n=40); 75% of these will be eligible on remaining criteria (n=30) and 33% (n=10) will consent to participate, per quarter. Based on our past experiences, this consent rate is feasible [51, 52].

A. The Royal Women's Hospital

1. The oncology trials research (OTR) team member will identify women with Stage I, II or III uterine, cervical, fallopian tube, primary peritoneal or ovarian cancer or BOT who may be suitable to participate from the clinic list. This identification/'early flagging' process may occur at several different time-points:
    a. at outpatient clinics prior to surgery
    b. pre-operatively on day of surgery,
    c. at the 6 to 8-week post-surgical check,
    d. at the 3-month post-surgical check (if adjuvant therapy administered).
2. The OTR team member will provide potentially eligible women a study brochure and obtain their consent to be contacted by the research team.
3. The OTR team member will complete an "Eligibility Checklist" for all women approached which they will scan and email to a research team member.
4. A research team member will contact potentially eligible women who consent to be contacted, via phone within 1-2 weeks, to discuss the study in more detail.
5. For those interested in taking part the research team member will:
    a. check initial eligibility by completing the 'Eligibility Screening Form',
    b. obtain consent to contact them again ≥ 6 months after completion of primary cancer treatment, or ≥ 3 months after completion of adjuvant therapy
    c. send a follow-up email containing the PICF and confirming a date for a follow-up call and potential study enrolment.
6. At least 6 months after completion of primary cancer treatment or ≥ 3 months after completion of adjuvant therapy and within a week of the scheduled follow-up phone call an authorised research team member will remotely check potentially eligible women's medical records for any changes to eligibility.
7. The research team member will then contact women who are still eligible, via telephone, at the date agreed upon. They will reconfirm eligibility and send the PICF and baseline questionnaire, via email or post (dependent on the woman's preference).
8. For women who are no longer eligible based on changes identified in the medical records, a research team member will contact them via telephone, at the date agreed upon and explain they are no longer suitable to take part in the study and thank them for their time.
9. The research team member will keep a record of all women approached to take part in the study and any reasons for women not taking part (e.g. not interested/did not meet inclusion criteria, met exclusion criteria).

10. In addition, patient medical records of all patients who consent to participate and complete baseline questionnaires will be reviewed by an authorised member of the research team to confirm eligibility for the study according to the inclusion and exclusion criteria, including tumour site and stage.

B. Other public hospital gynae-oncology outpatient clinics

1. A research team member will be onsite at the gynae-oncology outpatient clinic and identify women with Stage I, II or III uterine, cervical, fallopian tube, primary peritoneal or ovarian cancer or BOT who may be suitable to participate, from the clinic list. This may occur at several different time-points:
    a. at pre-operative appointments
    b. at post-operative appointments
    c. surveillance appointments
2. The research team member will attach a study brochure and "Eligibility Checklist" to potentially eligible women's case notes file.
3. Either the treating specialist or oncology nurse will provide the study brochure and discuss the study with potentially eligible women. They will also complete the 'Eligibility Checklist' and obtain the woman's consent to be contacted by the research team via telephone within 1-2 weeks or in person on that day, if a research team member is onsite.
4. The treating specialist or oncology nurse will give all completed Eligibility Checklists to the research team via email or in person.
5. When the research team member makes contact with the potentially eligible woman, they will discuss the study in more detail and for those interested in taking part the research team member will
    a. check initial eligibility by completing the 'Eligibility Screening Form',
    b. obtain consent to contact them again ≥ 6 months after completion of primary cancer treatment or ≥ 3 months after completion of adjuvant therapy, and
    c. send a follow-up email containing the PICF and confirming a date for the follow-up call and potential study enrolment.
6. At least 6 months after completion of primary cancer treatment or ≥ 3 months after completion of adjuvant therapy and within a week of the scheduled follow-up phone call an authorised research team member will remotely check potentially eligible women's medical records for any changes to eligibility.
7. The research team member will then contact women who are still eligible, via telephone, at the date agreed upon. They will reconfirm eligibility and send the PICF and baseline questionnaire, via email or post (dependent on the woman's preference).
8. For women who are no longer eligible based on changes identified in the medical records a research team member will contact them via telephone, at the date agreed upon and explain they are no longer suitable to take part in the study and thank them for their time.
9. The research team member will keep a record of all women approached to take part in the study and any reasons for women not taking part (e.g. not interested/did not meet inclusion criteria, met exclusion criteria).
10. In addition, patient medical records of all patients who consent to participate and complete baseline questionnaires will be reviewed by an authorised member of the research team to confirm eligibility for the study according to the inclusion and exclusion criteria, including tumour site and stage.

C. Private gynaecology clinics (if deemed necessary to reach recruitment targets)

1. Potentially eligible women (those receiving treatment for Stage I, II or III uterine, cervical, fallopian tube, primary peritoneal or ovarian cancer or BOT) will be approached by a clinic staff member during outpatient appointments.
2. The clinic staff member will provide the community study brochure and discuss the study with potentially eligible women.

3. The potentially eligible women will self-initiate contact with the study, by using a REDCap QR code listed on the brochure. Women will complete online questions regarding inclusion and exclusion criteria by, and provide agreement to be contacted by a research team member by phone.
4. A research team member will contact eligible women within 1-2 weeks.
5. During this call, the research team member will discuss the study in more detail and for those interested in taking part the research team member will:
    a. check initial eligibility by completing the ‘Eligibility Screening Form’, and ensure woman is ≥ 6 months after completion of primary cancer treatment or ≥ 3 months following completion of adjuvant therapy. If not, obtain consent to contact woman again once she becomes eligible
    b. send a follow-up email containing the PICF and confirming a date for the follow-up call and potential study enrolment.
6. Once the consent form has been signed, a research team member will send the baseline questionnaire, via email or post, dependent on the woman’s preference.
7. The research team member will keep a record of all women approached to take part in the study and any reasons for non-consent.
8. In addition, patient medical records of all patients who consent to participate and who have completed baseline questionnaires will be reviewed by a different member of the research team to confirm eligibility for the study according to the inclusion and exclusion criteria, including tumour site and stage. If the patient medical records are not held at one of the three main hospital sites (Monash Health, RWH & Mercy Hospital for Women), the patient will be asked to provide written evidence of tumour site/staging and treatment from a medical report. If the patient is unable to produce this evidence, with the patient’s consent, a research team member will send a template letter to the patient’s GP or specialist requesting confirmation of the woman’s cancer diagnosis and treatment.

D. Community via social media

1. An advertisement will be placed on community foundations/social media/care group web-sites or support groups associated with gynaecological cancer to attract potentially eligible women who have received treatment for Stage I, II or III uterine, cervical, fallopian tube, primary peritoneal or ovarian cancer or BOT.
2. Potentially eligible women will complete questions regarding inclusion and exclusion criteria via a REDCap survey link and provide agreement to be contacted by a research team member by phone.
3. *For next steps of recruitment process please refer to Section C, steps 4-8, above.*

All women will provide informed consent to participate using either an online consent form within REDCap^{TM} or paper-based forms and returned via email or post (depending on the woman’s preference). Consent will be obtained prior to the completion of any baseline assessments. Baseline, 17 and 52-week assessments will be collected remotely via online surveys using REDCap^{TM} software or paper-based and returned via email or post (depending on the woman’s preference).

5.2 Eligibility Criteria

5.2.1 Inclusion Criteria

- Aged 18 years or above
- Women following cancer treatment (with and without radiotherapy) for Stage I, II or III uterine, cervical, fallopian tube, primary peritoneal or ovarian cancer or BOT;

- Ability to speak and read English sufficiently for purposes of the study
- Primary cancer treatment completed ≥ 6 months ago or adjuvant therapy completed ≥ 3 months ago
- Self-reported urinary incontinence (≥ 1 episode per week for last 4 weeks);
- Have not received >1 physiotherapy-supervised pelvic floor treatment for urinary incontinence (in clinic or telehealth) since commencing cancer treatment or in the previous 4 years (whichever is most recent); and
- Have a home internet connection and a smartphone.

5.2.2 Exclusion Criteria

- Pregnant or breastfeeding;
- Have given birth within the last 12 months;
- Have a severe pelvic organ prolapse;
- Have a vaginal pessary ring in situ;
- Have a significant neurological disorder;
- Have a severe physical or psychiatric impairment;
- Have had pelvic surgery for incontinence or pelvic organ prolapse in the last 2 years.
- Not willing to use an intra-vaginal biofeedback device or videoconferencing software (Zoom or other).
- Unable to give informed consent or complete all study and assessment procedures (including the e-health components of the study).

Figure 1: Summary of recruitment procedures

	Participant **identification**	Initial **eligibility + consent** for RT contact	**Eligibility** confirmation + **study details** provision	Follow-up + **full eligibility screen** ≥ 6 months post-treatment
the women's the royal women's hospital victoria australia	By the OTR[1] from the clinic list @<br>1. outpatient clinics prior to surgery and/or<br>2. pre-operatively on day of surgery and/or<br>3. post-surgical check	▪ OTR will provide brochure & complete "Eligibility Checklist" & send to RT[2]	RT will contact consenting women<br>▪ discuss study<br>▪ complete 'Eligibility Screening Form'<br>▪ send PICF[3]<br>▪ schedule follow-up call for ≥ 6 months post-treatment	RT will call potentially eligible women<br>▪ re-confirm eligibility<br>▪ Send PICF, consent & baseline questionnaire
Western Health	By the onsite RT from the clinic list @ treatment planning stage	▪ RT will affix brochure & "Eligibility Checklist" to case notes<br>▪ Specialist/nurse will complete "Eligibility Checklist" & provide to onsite RT		
Mercy Health	By the onsite RT from the clinic list @ treatment planning stage	▪ RT will affix brochure & "Eligibility Checklist" to case notes<br>▪ Specialist/nurse will complete "Eligibility Checklist" & provide to onsite RT		
Monash Health	By the onsite RT from the clinic list @ treatment planning stage	▪ RT will affix brochure & "Eligibility Checklist" to case notes<br>▪ Specialist/nurse will complete "Eligibility Checklist" & provide to onsite RT		
Private Clinics	By the clinic nurse:<br>1. post-operatively OR<br>2. during outpatient appointment.	Clinic nurse will provide brochure & complete "Eligibility Checklist" & send to RT		
Volunteer Database	RT will extract details of participants from previous studies who consented to be contacted again			

1 = oncology trials team member (OTR); 2=Research Team (RT); 3=Participant information and consent form (PICF)

5.3 Informed Consent Process

All participants will receive verbal and written information about the purposes, potential risks and processes involved in the study from a member of the research team. In accordance with the latest revision of the World Medical Association Declaration of Helsinki, informed consent will be obtained from all participants by signing the consent form after understanding the information delivered.

In the first instance, potentially eligible women who consent to be contacted by the research team, will be contacted by a research team member. Contact may be over the phone or in person, if a member of the research team is present at the clinic, at the time. This discussion will involve a verbal description of the project to ensure that participants are happy to comply with trial procedures and any questions will be answered (see example 'Participant Eligibility Script's). During this discussion, those expressing interest in being involved will be assessed for potential eligibility by the research team member (see 'Eligibility Screening Form'). Potentially eligible women meeting the eligibility criteria, will then be provided with the Participant Information and Consent Form (PICF) either in person, in the post or via email (dependent on availability and preference). Potentially eligible women who have completed treatment ≥ 6 months or ≥ 3 months after completion of adjuvant therapy ago will be offered a follow-up phone call within a week to discuss the study further. Potentially eligible women who have completed treatment within the last 6 months or ≥ 3 months after completion of adjuvant therapy will be offered a phone call at a date after they have completed treatment ≥ 6 months ago or ≥ 3 months after completion of adjuvant therapy. All potentially eligible women will be encouraged to phone researchers if they have any questions or concerns regarding the contents of the PICF before their next contact with the research team.

After reading the PICF, those wishing to participate can consent in two ways. Consent will be obtained either i) online via a survey (REDCap software) or ii) in hard copy by signing the paper-based consent form and returning it in a reply-paid envelope in the post or by scanning and emailing the document to a research team member. In addition, consent will be re-confirmed verbally by the treating physiotherapist prior to each telehealth-delivered consultation.

5.4 Enrolment and Randomisation Procedures

Participants will be enrolled into the study once the informed consent process has been completed and they have completed the baseline questionnaires on the web-based platform (REDCap) or via post (dependent on preference). Those who prefer to receive the baseline questionnaire via post will be asked to send the completed questionnaire back to a research team member in a reply-paid envelope or by scanning and emailing the document.

Once returned, baseline questionnaires will be checked by the research team for completeness and to confirm relevant eligibility criteria are still met (e.g. self-reported urinary incontinence frequency). Once this is confirmed, participants will be randomised (in permuted random blocks stratified by radiotherapy or not) into one of two groups. The randomisation schedule will be created by an independent statistician to ensure study statisticians remain blinded to treatment allocation and stored on a password-protected website (REDCap) at the University of Melbourne, maintained by a researcher not involved in either participant recruitment or administration of primary/secondary outcome measures. Group allocation will be revealed by a different member of the research team, who has had no contact with participants. This person will notify the research assistant of the assignment, and the research assistant will contact the participant, via the telephone, after baseline assessment has been completed. Each participant will receive a unique study ID code, and this will be documented in the participant's record/database in addition to all study documents.

5.5 Blinding Arrangements

As this is a pragmatic trial and as is common to exercise interventions, participants will not be blinded to group allocation. Therefore, as the primary and secondary outcomes are participant-reported, by default the assessors of these outcomes (the participants) are not blinded. Members of the research team administering and entering participant-reported data will be blinded. The statistical analysis plan will be developed by a biostatistician blinded to group allocation and published prior to unblinding and statistical analysis.

5.6 Participant Withdrawal

As participation in this study is voluntary, women may withdraw at any point during their involvement. All participant withdrawals will be recorded including the nature, timing of and reasons for withdrawal (provided the participant responds to contact made by the research team). Data will be analysed on an intention-to-treat basis. Sensitivity analyses will be conducted to investigate differences between those who withdraw and those who provided full data.

A participant may choose to withdraw fully or partially from their involvement in the study. Should a participant choose to withdraw fully from the study no further information will be collected. The participant will be asked if the data collected prior to their withdrawal is able to be used. Should they decline, their data will be removed from the database and stored in a separate folder to ensure it is not included in the final analysis. Participants may also choose to withdraw partially by ceasing their tele-health consultations but continue with some/all aspects of data collection. In order to minimise data loss, research staff will encourage participants to complete the primary outcome measure, at the 17-week time frame, over the telephone if necessary.

5.7 Trial Closure

For each participant, the longest follow-up duration will be 52-weeks from randomisation. After this point no further data collection or monitoring will occur.

5.8 Continuation of therapy

Intervention group participants are permitted to keep their femfit® biofeedback device and continue to use the associated smartphone app after their involvement in the study has ended. Therefore, participants can choose whether they wish to continue their PFMT program or not.

6. STUDY VISITS AND PROCEDURES SCHEDULE

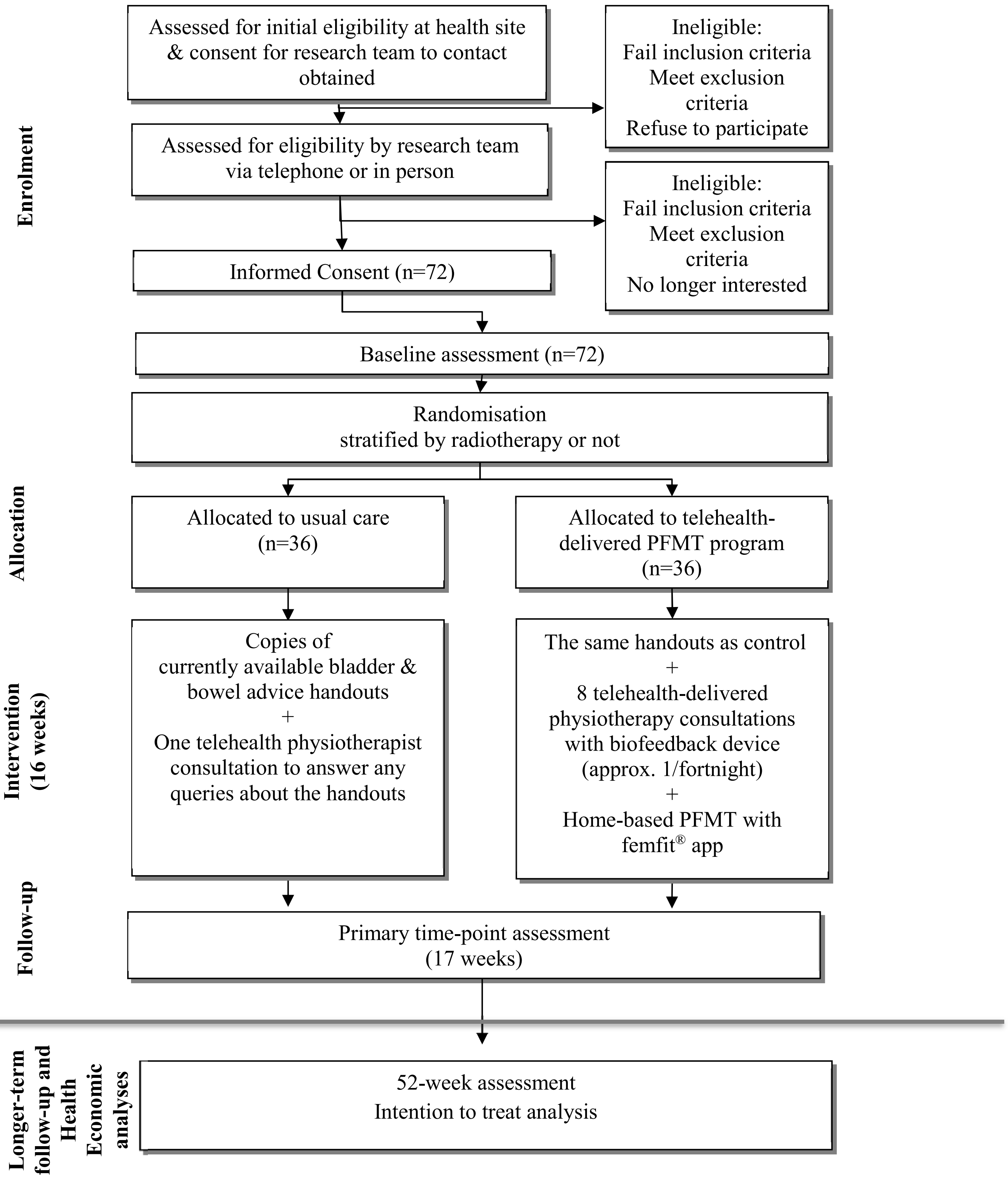

7. DATA COLLECTION

All outcome measures are participant-reported and will be collected electronically via computer within REDCap software or in hard copy with paper and pen (if preferred). If hard copies are completed responses will be entered into the study computer database by a research team member. It is estimated that the full baseline assessment questionnaire will take approximately 30-45 minutes to complete, and follow-up assessment questionnaires will take approximately 15-25 minutes to complete. Participant accident diaries will be completed in hard copy or online (dependent on preference) at all time points. Participants will be expected to return completed accident diaries to the research team, via post, in a provided reply-paid envelope or by scanning and emailing, at baseline, 17-weeks, and 52 weeks. It is estimated that completing each accident diary will take 5 minutes in total.

8. INTERVENTIONS

8.1 Usual care (Control)

Participants in this group will receive bladder and bowel advice handouts currently available from the Continence Foundation Australia and the Australian Government Department of Health (see 'Example Control Group Resources' document). The research team member will send the handouts via email or post (dependent on the woman's preference), directly after randomisation. Also, after randomisation the research team member will schedule one, (up to) 15- minute tele-health consultation (via telephone) with a research team member, 1-2-weeks after completion of baseline assessments. During this telephone call, the researcher will check the handouts have been received, answer any handout or study related queries and remind the participant of the dates of their follow-up assessments. The researcher will not provide any tailored PFM advice/instruction.

8.2 Telehealth-delivered pelvic floor muscle training program (Intervention)

Participants in the intervention group receive the same handouts as the control plus an intensive 16-week physiotherapist-supervised telehealth-delivered PFMT intervention. Pelvic floor muscle exercise will be supported by a Therapeutic Goods Administration (TGA) approved femfit® biofeedback device[53]. Directly after randomisation, a research team member will schedule each participant for their first telehealth-delivered physiotherapy consultation. The aim will be for the initial consultation to be within 2 weeks of baseline assessments. Subsequent tele-health appointments will be made by the treating study physiotherapist in consultation with the woman. Prior to a participant's first physiotherapy consultation, a member of the research team will schedule and conduct a teleconference consultation with the participant to practise the technical aspects of internet connection and screen sharing (approximately 15 – 30 minutes). Twenty-four hours prior to the participant's first telehealth-delivered physiotherapy consultation, a member of the research team will send a consultation reminder via mobile phone text message to the participant.

Study physiotherapists
We will recruit 3 physiotherapists from our study sites and/or the broader community to deliver the tele-health consultations for both the pilot study and the RCT. Physiotherapists will be eligible if they i) have postgraduate training in pelvic floor physiotherapy; ii) are able to commit to accepting at least one new study participant for their per-protocol intervention each week; iii) agree to comply with all study processes and procedures. Advertisements will be circulated through our study sites and community-based women's health physiotherapy clinics. Eligibility will be assessed via response to a University of Melbourne Position Description. Physiotherapist will be paid for their involvement in

this pilot study/preliminary research. These physiotherapists will have participated in our pilot study (see 9.1.1).

Physiotherapists in the RCT will treat approximately 12 patients each. Study physiotherapists will receive a detailed study manual and will complete training in study processes and procedures (Refer to Table 1). Within the study manual, a 'consultation notes' template will be included and used to document each participant consultation. Physiotherapists can choose to complete consultation notes electronically or on paper, depending on their preference. Consultation notes will be returned to the research team, by the physiotherapist, at the completion of each participant's course of tele-health consultations.

Co-design of study intervention and study resources
Prior to commencing this trial, we will conduct preliminary research (a Pilot study) with the three recruited study physiotherapists and three women who have undergone gynaecological cancer treatment (including at least one woman with past radiotherapy treatment) to co-design all physiotherapist and participant resources and intervention procedures. We feel three women will be sufficient to test the Zoom exercise instruction procedures required for the subsequent RCT. In addition, we have further feedback from women with cancer who are participating in single-arm feasibility studies that we are currently undertaking (scheduled to complete before our RCT commences), to supplement the feedback we will receive from this pilot study. Specific detail of intervention components will be included in this protocol as they are developed and finalised.

Patient resources
After randomisation and before their first tele-health consultation each intervention group participant will receive by registered post:

- A participant study manual that will include:
    - a welcome letter describing their involvement in the study and listing all study resources that they will have received
    - detailed instructions of how to use the femfit® biofeedback device and associated smartphone app
    - detailed instructions of how to use the tele-health Zoom Cloud Meetings software (Zoom) or other teleconferencing platform of choice for physiotherapy consultations
- information regarding good bladder and bowel health (same as control group)
- participant diaries (e.g. exercise and accident diaries)
- a femfit® biofeedback device.

Physiotherapist-supervised tele-health consultations
Each woman will be randomly allocated to one of the three study physiotherapists, according to a randomisation schedule supplied by the statistician. Women will receive 8 consultations with one study physiotherapist, via teleconference software.
Study physiotherapists will be provided a PDF copy of each baseline questionnaire of participants allocated to them. This will be sent by the research team via email and prior to a participant's initial consultation. This is in order to facilitate discussions between the physiotherapist and women about past medical history and current symptoms at the first consultation, and to save participant time and burden explaining history and symptoms that she has already explained in the baseline questionnaire. Baseline questionnaires will not contain participant names and will only contain participant study codes. Each consultation will be approximately 30-60-minutes duration and contain both education and exercise components (estimated consultation duration: initial 55-60 minutes; subsequent 30 minutes; final 45 minutes). The same physiotherapist will complete all consultations with a participant, unless there are unforeseen circumstances (e.g. physiotherapist illness). In this instance, a research team member will arrange for another study physiotherapist to conduct any required future consultations. At the start of every consultation, and prior to use of the femfit® device the physiotherapist will review the femfit® contraindications of use. Contraindications are: i) menstruating (the device cannot be inserted but the woman can still use the app) and ii) vaginal infection. In addition, physiotherapists will be asked to record three consultations (consultation 1, 4 and 8) with consenting participants and provide the audio

files to the research team. Audio files will be saved on secure, password protected University of Melbourne servers. The research team will conduct random audits of audio files to evaluate intervention fidelity throughout the trial. Physiotherapists will only record consultations with women who consent to having their consultations recorded for intervention fidelity monitoring purposes.

The components of the consultations are described in Table 1 according to the 16-items of the CERT-PFMT [41]. The CERT-PFMT are the recommended reporting guidelines to inform how to document pelvic floor muscle training to enable replication in clinical practice.

Table 1 Components of the physiotherapist-supervised tele-health consultations

CERT PFMT item	Description
1. Exercise equipment	femfit® biofeedback device to help teach the correct PFM contraction technique, to monitor PFM strength gains and facilitate PFMT adherence.
2. Instructor qualifications	Professional qualification: physiotherapist with postgraduate training in pelvic floor physiotherapy.<br><br>Per-protocol study training: detailed study manual and training in study processes and procedures including:<br>- pelvic floor muscle assessment and exercise instruction processes;<br>- instructions for teaching use of the biofeedback device;<br>- biofeedback data export;<br>- practice video-consultations to practice video-consultation skills.<br><br>During the study: regular meetings with the research team to review per-protocol procedures and discuss/address potential issues as they arise.
3. Individual or group	Individual physiotherapist supervised telehealth-delivered instruction in PFMT and individual home-based PFMT.
4. Supervision	Supervision will be provided remotely by a trained physiotherapist via 8 telehealth-delivered consultations, over 16 weeks. Between tele-health consultations woman will complete their PFMT unsupervised.
5. Adherence	Adherence to PFMT will be reported within the femfit® App.<br><br>Attendance at physiotherapy consultations will be recorded by the physiotherapist.
6. Motivation	Biofeedback will be provided via the femfit® device and associated app.<br><br>In addition, an extensive list of motivational strategies that may be suggested by the physiotherapist to aide PFMT will be formulated in our preliminary research*.<br><br>The finalised list of strategies/prompts will be added to this protocol prior to commencement of recruitment. Example strategies may include goal setting, specific instructions/wording on how to perform PFMT, verbal persuasion, visual cues.
7. Progression decision rules	The femfit's ® pre-determined, exercise program and recommended progressions will be used which are evidence-based and clinically validated [54].<br><br>Within the femfit® app the intensity and repetitions of the exercises increase every 4 weeks. In the first month, the exercise program will take around 8 minutes to complete daily. This increases to 12 minutes in the second month, 16 minutes in months 3 and 4 and for the remainder (maintenance phase). The position to do the exercises also changes from lying to supported standing, to standing.<br><br>Preliminary research will inform any required adjustments to exercise progression guidelines*.

8. Description of each exercise (including teaching strategies)	Pelvic floor muscle exercises included in the femfit ® are: maximal contraction, co-ordination, endurance, contraction with cough. These will be described in the femfit® app as "Squeeze", "Rapid", "Enduro", and "Knack".<br><br>The specific exercise program will be adjusted as required for this intervention in consultation with the recruited study physiotherapists. This will be achieved via consensus of the optimal standard face-to-face pelvic floor exercise assessment and instruction processes. Once developed, any additional detail will be included within this protocol.
9. Home program	PFM exercises, as prescribed by the physiotherapist, will be conducted at home. Specific home exercise details regarding position, time, dosage, effort and use of the femfit® app will be provided by the physiotherapist at each consultation.
10. Non exercise components	Intervention participants will receive the same informational handouts regarding good bladder and bowel health as the control group.<br><br>Additional information will be provided as indicated:<br>- For urinary urgency and frequency or urge urinary incontinence: bladder training including urinary urge suppression techniques, education on fluid intake modification including caffeine reduction if relevant, and education in good bladder and bowel habits.<br>- For faecal incontinence: bowel routine training, education on dietary fibre including psyllium supplementation, education on faecal urgency suppression techniques.<br><br>Preliminary research will inform the development of the educational information provided*.
11. Adverse events	Refer to section 11 of this protocol.
12. Setting	Tele-health and HEP will be conducted in women's homes.
13. Dosage	Refer to details provided in Item 8. Description of each exercise (including teaching strategies).
14. Generic or tailored	The femfit's® pre-determined, exercise program will be used which is evidence-based and clinically validated [54]. Additional tailoring to each woman may be recommended by the treating study physiotherapist based on assessment findings.<br>Preliminary research will inform exercise tailoring guidelines*.
15. Starting level assessment	The femfit's® pre-determined starting level will be used. The starting level may be modified by the treating study physiotherapist based on assessment findings.<br>Preliminary research will inform exercise starting level modification guidelines*.
16. Intervention fidelity	Consultation notes will be reviewed and analysed by the research team to assess the physiotherapists' adherence to trial protocols.<br>The research team will also conduct random audits of audio recorded consultations.<br>Both the consultation notes template and the specific criteria that will be applied to the consultation notes to evaluate fidelity will be developed in preliminary research (Pilot study) in consultation with the recruited physiotherapists.

* Preliminary research: A pilot study will be conducted including consultation and co-design of resources and intervention processes and procedures with the recruited study physiotherapists (n=3) and women who have had gynaecological cancer treatment (n=3). All study materials will be created specifically for this intervention.

9. ASSOCIATED PROCEDURES/STUDIES

Our entire research project is made up of three studies, as illustrated below.

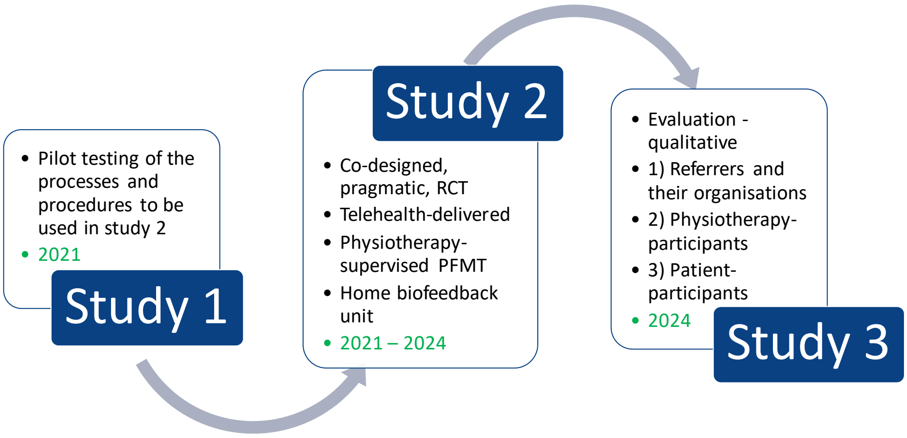

9.1 Piloting of the intervention

Study 1: Prior to commencing the RCT, we will conduct pilot testing of the processes and procedures to be used in the intervention arm of this RCT in order to 'road-test' and refine the intervention details. This will be achieved through a co-designed action and consumer-participatory study. We will recruit the physiotherapists who will deliver the intervention for both the pilot study and the RCT (n=3), and patient-participants (n=3), who have had gynaecological cancer. We will apply an iterative design and will test the components of the intervention from both physiotherapy and participant perspectives. This pilot study is included in the main trial's ethics application and has its own recruitment and consenting procedures.

<u>Physiotherapy-participants</u>

Recruitment: Three physiotherapists will be recruited to take part in the pilot study and the RCT. Physiotherapists will be recruited from the community via advertisements and will be contracted by the University of Melbourne to delivery physiotherapy services for the intervention in the clinical trial. All study physiotherapists will be invited to participate in the pilot study to assist in co-designing and developing the intervention procedures for the clinical trial. Study physiotherapists will receive a PICF statement outlining what the pilot study involves and have a discussion with a researcher to ensure they understand what participation involves. If they decide to be involved, they will complete a consent form prior to their involvement.

Physiotherapist-participant's role in pilot study/preliminary research: the recruited and consenting study physiotherapists will be involved in the iterative review and refinement of processes for the clinical trial. The physiotherapists will receive a draft study manual and will participate in a 60-90 minute focus group. Physiotherapists will complete a consent form prior to their involvement in the focus group.

The focus group will involve:
i) consensus development of the key elements of face-to-face PFMT and exercise instruction (structured according to the 16-items of the CERT- PFMT [41]),
ii) review of draft consultation processes (e.g. use of zoom, use of the femfit® device and data export, consultation note keeping and preferences for receiving participant information prior to the initial consultation (or not), will be explored).
iii) a review of administrative 'housekeeping' for the clinical trial (e.g. clinical trial background information, processes for taking leave, how to bill for consultations.

After the focus group, the research team will update the draft study manual incorporating insights from the focus group. Each physiotherapist will receive the updated study manual and will then be scheduled to conduct three practice video-consultation (an initial, subsequent, and final consultation) with patient-participants to practice their video-consultation skills and road test intervention procedures. Physiotherapists will be asked to record all practice-consultations and provide the audio files to the research team. Audio files will be saved on secure, password protected University of Melbourne servers. Audio files will be reviewed by a member of the research team for training purposes. After completing their practice consultations, a 30-60-minute meeting will be organised with all three study physiotherapists and the research team to finalise study procedures prior to the RCT commencing recruitment.

Patient-participants

Recruitment: We will recruit three women (at least one who has undergone radiotherapy) to participate in the pilot study/preliminary research. The principal investigator will invite women to participate who are already known to the researchers through their prior involvement in providing consumer feedback or as participants in our previous research who have consented to be contacted about future research. Initial contact will be via an email invitation which will contain the PICF statement outlining what the pilot study involves. Women who respond to the invitation by reply email saying that they may be interested in participating will be contacted by the research team to discuss the pilot study in more detail. Women will be told they can take as long as they need to make a decision regarding participation. If they decide to be involved they will be asked to complete a consent form prior to their involvement. Their commitment is estimated to be no greater than 3 hours in a week and for a maximum of 4 months. Patient-participants will receive a $20 gift voucher in gratitude for their time commitment.

Patient-participant's role in pilot study/preliminary research: recruited patients will help with road-testing procedures for the clinical trial both in person and over Zoom.
This will include piloting internet connection/Zoom instructions; informing the need for participant digital technology up-skilling which may be required to optimally benefit from telehealth consultations; completion of online and paper-based participant-reported outcome measures; use of the biofeedback device and in-person validation of insertion instructions. Patient participants will also provide detail regarding the time taken to complete all study-related procedures and will conduct practice video-consultations with recruited physiotherapist-participants. Patient participants will provide input in a variety of ways including via email, in person and/or 1:1 phone calls with members of the research team and through participation in practice-consultations with study physiotherapists.

9.2 Qualitative evaluation

Study 3: Qualitative data will be collected through telehealth-conducted interviews using a semi-structured interview guide formulated by the researchers and consumer collaborators to explore referrer, physiotherapy and participant experiences of the intervention. Interviews will be audio-recorded, transcribed and analysed using a Grounded Theory framework. The qualitative study will be reported separate to the main trial (Study 3 in figure above) and will have its own ethics application and consent procedures.

9.3 Cost effectiveness analysis

We will also follow-up all participants at 52-weeks. We will ask them to complete all measures. This additional assessment will test any maintenance in treatment effect, and a cost effectiveness analysis if clinical effectiveness is demonstrated.

10. PARTICIPANT SAFETY

10.1 Risk Management and Safety

We anticipate no substantial risks nor safety concerns associated with participation in this study. Intervention group participants will be required to use the femfit® biofeedback device during their tele-health consultations and during their prescribed PFM HEP. The femfit® is a low-risk (class I) device which has undergone extensive electro-magnetic compatibility, biocompatibility and electrical safety testing and has been approved by the TGA[53]. Information regarding the use of an intra-vaginal biofeedback device will be included in all recruitment processes (study discussions with the research team member, information within the study). This will ensure women are aware that if randomised to the intervention group they will be asked to use this device and are therefore able to provide informed consent. Women randomised into the intervention group will receive detailed instructions (written in their study manual and verbal from the physiotherapist during their tele-health consultations) about how to insert and remove the femfit® sensor. As the femfit® sensor is smaller than an index finger, it is not anticipated that insertion of the sensor into the vagina would cause any discomfort to the woman. However, if an intervention group participant, during their involvement in the trial, experiences discomfort or distress using the femfit® device they will be asked to discuss this with their treating study physiotherapist and use of the femfit® may be discontinued.

We recognise that for some women receiving an invitation to participate in this study or completing the questionnaires may cause distress. If a woman experiences distress as a result of the invitation to participate or actual participation in the study, they will be directed to contact the Chief Investigator (A/Prof Helena Frawley), in the PICF and participant study manual. The Chief Investigator will discuss with the woman the nature and severity of the distress and will determine what further action is required. Further action may include a request to contact their General Practitioner as soon as possible, withdrawal from part or all of the study intervention or withdrawal from the study. If deemed necessary additional action may also include:

- Review in the gynae-oncology clinic with a consultant gynae-oncologist
- Referral to psychology/psychiatry services at the referring health service, or in the local community, as appropriate
- Linking the woman with the health service consumer advocate
- Acute mental health assessment by the CAT team.

11. ADVERSE EVENT REPORTING

Due to the nature of the intervention, any risks to participants are likely to be minor and transient. The reporting and handling of all adverse events (AE) will be in accordance with NHMRC guidelines[55], which defines an AE as: “Any untoward medical occurrence in a patient or clinical trial participant administered a medicinal product and that does not necessarily have a causal relationship with this treatment” and a serious AE as any AE that: results in death; is life threatening; requires inpatient hospitalisation or prolongation of existing hospitalisation; results in persistent or significant disability/incapacity; is a congenital anomaly/birth defect. Due to the low-risk nature of the interventions in this trial, serious AE are extremely unlikely but will be reported to the primary Human Research Ethics Committee should they occur. We will record all adverse events, including minor, expected or unexpected (as described in the PICF), according to the NCI-CTCAE(v5) criteria [57]:

- Grade 1 Mild; asymptomatic or mild symptoms; clinical or diagnostic observations only; intervention not indicated.
- Grade 2 Moderate; minimal, local or non-invasive intervention indicated; limiting age-appropriate instrumental activities of daily living
- Grade 3 Severe or medically significant but not immediately life-threatening; hospitalization or prolongation of hospitalization indicated; disabling; limiting self-care activities of daily living

- Grade 4 Life-threatening consequences; urgent intervention indicate
- Grade 5 Death related to AE

Information regarding AE may be reported by participants in the 17 and 52-week follow-up assessment questionnaires in section E: Health problems. Adverse events may also be reported by the woman directly to the treating study physiotherapist or to a member of the research team. Any AE reported to the study physiotherapist will be recorded in the consultation notes and if serious will be reported to the research team to follow-up and action. Any AE reported to the research team will be either i) recorded in a custom-built 'Adverse Events Form' and then entered into a "Participant Adverse Event" excel spreadsheet or ii) entered directly into the excel spreadsheet (see 'Standard Operating Procedure: Adverse Event Reporting' document). This process will include the member of the research team ascertaining and subsequently recording the likelihood that the reported AE is related to the intervention. This will be done according to the NCI Guidelines for Investigators [58]:

Relationship	Attribution	Description
Unrelated to investigational agent/intervention	Unrelated	The AE is clearly NOT related to the intervention
	Unlikely	The AE is doubtfully related to the intervention
Related to investigational agent/intervention	Possible	The AE may be related to the intervention
	Probably	The AE is likely related to the intervention
	Definite	The AE is clearly related to the intervention

All AE will be reported to the internal Trial Advisory and Safety Committee who will be responsible for deciding what action if any is needed on a case-by-case basis.

12. STATISTICAL METHODS

12.1 Sample Size Estimation

We aim to detect a minimal clinically important difference (MCID) of 2.5 units [35] over 17 weeks in the primary outcome of change in urinary incontinence measured using the ICIQ-UI SF[34]. The sample size calculation accounts for potential clustering by physiotherapists in the intervention arm. Based on previous research[35], we assume a conservative between-participant standard deviation of 3.2 units. We have also assumed a conservative correlation between baseline and 17-week scores of 0.4, an intra-cluster correlation of 0.05 and 3 physiotherapists treating approximately 10 patients each. With these parameters, we need 30 women per arm to achieve 80% power to detect the MCID at a 0.05 significance level. Allowing for 15% attrition, we will recruit 36 women per arm (in total n=72).

12.2 Statistical Analysis Plan

All analyses will be described *a priori* in a detailed Statistical Analysis Plan and published while biostatisticians are blinded to treatment allocation. Demographic and baseline characteristics of participants will be summarised as appropriate (means and standard deviations for continuous variables that appear to be distributed approximately symmetrically, medians and interquartile ranges for other continuous variables, counts and percentages for categorical variables). Main comparative analyses between groups will be performed using intention-to-treat. Constrained longitudinal data analysis will be used to analyse continuous outcomes, including the primary outcome. The response will consist of all continuous outcomes (baseline, 17 weeks and 52 weeks) and the model will include factors representing treatment group, time (categorical), and a group-by-time interaction, with the restriction of a common baseline mean across treatment groups. Models will include the stratification

variable (radiotherapy) and random effects for physiotherapist (intervention arm only). The mean change in urinary incontinence (primary outcome) and other continuous outcomes (ICIQ-LUTSqol, PFBQ, EQ-5D-5L, UTAUT-II domains 1-9, METminutes (walking, moderate-intensity activities, vigorous-intensity activities) from baseline to each follow-up time-point between the two intervention groups will be obtained. The primary hypothesis will be evaluated by obtaining the estimated differences between the two intervention arms in mean change in urinary incontinence score from baseline to 17-weeks post-commencement of treatment (primary time point), two-sided 95% confidence intervals and p-values. These models provide valid inference in the presence of missing data if the data are missing at random. Standard diagnostic plots will be used to check model assumptions. Treatment effects at 52 weeks post-randomisation (secondary time point) will be estimated to assess maintenance of treatment effects for all outcomes except UTAUT-II domains 1-9 for which only 17-week follow-up data will be collected.

The binary secondary outcomes (PGIC and physical activity category) will be compared between groups separately using logistic regression, adjusting for the stratifying variable of radiotherapy, and fit using generalized estimating equations to account for clustering, with results reported as risk ratios and risk differences. Poisson regression models fitted using generalized estimating equations will be used for secondary count outcomes (number of leakage episodes overall and by severity and provocation, number of continence pads used).

The economic evaluation will have a societal perspective and will assess both the cost of the intervention at 17 weeks and the cost-effectiveness and cost-utility of the intervention group *versus* the control group at 52-weeks. Quality-adjusted life years (QALYs) gained [56] for the intervention compared to control at 52 weeks will be assessed. QALYs will be calculated based on utility scores using the EQ-5D-5L [44, 45] at baseline and 52 weeks. QALYs will also be calculated using the ICIQ-LUTSqol [42], a condition specific quality of life measure, at baseline and 52 weeks. Analysis will be combined with the primary clinical outcome measure to establish the incremental cost-effectiveness ratio of the intervention. The difference in participant resource use related to incontinence (e.g. pad use, medication use, health service use) and productivity lost between baseline and 52 weeks will be compared for intervention and control groups. The association between utility gains on the EQ-5D-5L and productivity will be compared between the intervention and control groups. The 52-week economic evaluation will be reported separate from the main trial.

12.3 Interim Analyses

It is not anticipated that any interim analyses will be performed.

13. DATA MANAGEMENT

13.1 Data Collection & Storage

13.1.1 Identifiable data

- Screening information and study consent forms will be stored within a website (REDCap) and accessible only by password to members of the researcher team. If women prefer to complete consent forms in hard copy, paper consent forms will be stored in locked filing cabinets, separate from a cabinet containing any de-identifiable data and only accessible to the researchers. They will also be scanned and stored in a computer database separate from any deidentified data.

- Details of all women screened will be stored electronically in a REDCap and/or Microsoft Excel database, accessible only to the research team and stored securely on password-protected servers.
- Clinical data from femfit® devices will be automatically stored within the third-party 'femfit server', via the femfit® app. The 'femfit® server' is a secure, password protected platform. Data stored within this secure server will be accessible only to the research team and to the treating study physiotherapists who will only access the data of their participants in order to review participant progress and adherence.
- Individually identifiable information will also be accessed in the form of patient medical records of patients who consent to be contacted for this study in order to confirm potential eligibility. Patient medical records of all patients who consent to participate and complete baseline questionnaires will also be reviewed to confirm cancer related medical history including tumour site and stage.
- Telephone contact details may also be obtained from patient medical records by a health professional member of their treating team and provided to the research team by this person. Patient contact details will only be collected following consent to contact being obtained from the patient. This information will be saved in a password-protected electronic document, stored electronically within secure password-protected servers.

13.1.2 Re-identifiable/coded data

- Questionnaires may be completed on paper or electronically, and will only contain participant study codes. No identifying information such as names, emails and postal addresses will be contained in the questionnaires. Paper copies will be stored in locked filing cabinets, separate from a cabinet containing any identifiable data and only accessible to the researchers. Electronic copies will be stored in the REDCap website, accessible only to the researchers by password protection. Data from within REDCap will be exported to Microsoft Excel and other statistical packages used by the researchers for analyses. These will be stored securely on password-protected servers.
- Copies of the Baseline questionnaires of women allocated to Group 2 (intervention) will be exported from REDCap into PDF format. PDF copies will be emailed to the allocated study physiotherapists by a member of the research team. Study physiotherapist will store PDF copies of baseline questionnaires on password protected computers saved with file names containing participant study codes only.
- For analysis clinical data from femfit® biofeedback devices will be exported from the femfit® server. Identifiable information will we removed, and data will be assigned a unique study code. Information with unique study codes will be stored electronically in excel spreadsheets accessible only to the research team and stored securely on password-protected servers.
- All computer files will be stored on secure and backed-up servers, accessible only to the researchers using a password.

13.2 Data Confidentiality

Each participant will be provided with a unique study identification number (study ID). Identifiable information that is collected in this study will only be accessible by research staff with security access. No information which could lead to the identification of a woman will be included in the dissemination of results. Only fully non-identifiable data will be presented when disseminating results. At no stage will a person's name or any identifying information be provided to a third party.

13.3 Study Record Retention

Data will be retained for 15 years consistent with clinical trial recommendations outlined in section 2.1.1 of the National Health and Medical Research Council's "Australian Code for the Responsible Conduct of Research". Computer based data will be deleted from secure computer servers and hard copies (e.g. paper-based questionnaires) will be disposed of via the University of Melbourne's confidential paper shredding and disposal service. Before the details from this study are destroyed, details will be entered into a databank, and will not be able to be linked to the women in this study. During the consenting process for the RCT woman will be asked to consent to have their de-identified details added to the databank for future ethically-approved research use related to investigation of pelvic floor disorders. Women who do not consent will not have any de-identified data added to the databank. Information in the databank will be able to be accessed by this research team or future research teams who undertake research related to pelvic floor function and dysfunction in women.

14. PROTOCOL DEVIATIONS

Any protocol deviations including errors applying inclusion/exclusion criteria and/or administration of the wrong intervention will be documented, if they occur, in the findings manuscript with the trial results (patient flow diagram/text).

15. ADMINISTRATIVE ASPECTS

The trial will be prospectively registered (ANZ Clinical Trials Registry) and the protocol published in a peer review journal.

15.1 Independent HREC approval

Ethics approval will be obtained from the Human Research Ethics Committee of Monash Health (for multi-site approval at RWH) and Mercy Hospital for Women.
University of Melbourne.

15.2 Participant reimbursement

Participants who complete their additional assessment (week 52) will receive a $20 gift card as a token of appreciation for their involvement in the study. Intervention group participants will also keep their femfit® device after their involvement in the study is complete (retail value $300). If intervention participants require videoconferencing equipment (e.g. a webcam) this will be provided to them by the research team and they will be able to keep this on completion of the study. If intervention participants are required to upgrade their internet plans to have their videoconferencing consultations, they will be reimbursed for any associated costs via gift cards.

15.3 Financial disclosure and conflicts of interest

Dr Kruger is the inventor of the femfit® device and is the CEO of the company that distributes the device. There are no other conflicts of interest to declare.

16. USE OF DATA AND PUBLICATIONS POLICY

We will publish a protocol paper prior to completion of the trial. The main trial will be published in an oncology or general medical journal. Statistical code may be made available from the statistician, upon

request from individual researchers. Data may be made available from A/Professor Frawley, upon request from individual researchers. In addition, the results of the trial will be disseminated through avenues such as conference presentations, professional organisations, media, social media and consumer organisations.

17. REFERENCES

1. Australia CFo. Continence in Australia a snapshot June 2019. 2109.
2. Ramaseshan AS, Felton J, Roque D, Rao G, Shipper AG, Sanses TVD. Pelvic floor disorders in women with gynecologic malignancies: a systematic review. Int Urogynecol J. 2018;29(4):459-76. doi: 10.1007/s00192-017-3467-4.
3. Abrams P, Smith AP, Cotterill N. The impact of urinary incontinence on health-related quality of life (HRQoL) in a real-world population of women aged 45-60 years: results from a survey in France, Germany, the UK and the USA. Bju International. 2015 Jan;115(1):143-52. PMID: WOS:000346651400026. doi: 10.1111/bju.12852.
4. Delancey JOL. The hidden epidemic of pelvic floor dysfunction: Achievable goals for improved prevention and treatment. Am J Obstet Gynecol. 2005;192(5):1488-95. doi: 10.1016/j.ajog.2005.02.028.
5. Hazewinkel MH, Sprangers MA, Taminiau-Bloem EF, van der Velden J, Burger MP, Roovers JP. Reasons for not seeking medical help for severe pelvic floor symptoms: a qualitative study in survivors of gynaecological cancer. BJOG. 2010 Jan;117(1):39-46. PMID: 19874292. doi: 10.1111/j.1471-0528.2009.02411.x.
6. Lindgren A, Dunberger G, Enblom A. Experiences of incontinence and pelvic floor muscle training after gynaecologic cancer treatment. Supportive Care in Cancer. 2017 Jan;25(1):157-66. PMID: WOS:000389354500020. doi: 10.1007/s00520-016-3394-9.
7. AIHW. Australian Burden of Disease Study: impact and causes of illness and death in Australia 2015—summary report. In: 21 ABoDSsnCnB, editor. Canberra: AIHW2019a.
8. Neron M, Bastide S, de Tayrac R, Masia F, Ferrer C, Labaki M, et al. Impact of gynecologic cancer on pelvic floor disorder symptoms and quality of life: an observational study. Scientific Reports. 2019 Feb;9. PMID: WOS:000459092800020. doi: 10.1038/s41598-019-38759-5.
9. Dumoulin C, Cacciari LP, Hay-Smith EJC. Pelvic floor muscle training versus no treatment, or inactive control treatments, for urinary incontinence in women. Cochrane Database of Systematic Reviews. 2018 (10). PMID: WOS:000449049600061. doi: 10.1002/14651858.CD005654.pub4.
10. Bø K. Physiotherapy management of urinary incontinence in females. Journal of Physiotherapy. 2020;in press. doi: https://doi.org/10.1016/j.jphys.2020.06.011.
11. Chuang T-Y, Yu K-J, Penn I-W, Chang Y-C, Lin P-H, Tsai Y-A. Neurourological changes before and after radical hysterectomy in patients with cervical cancer. Acta Obstet Gynecol Scand. 2003;82(10):954-9. doi: 10.1034/j.1600-0412.2003.00177.x.
12. Bernard S, Ouellet MP, Moffet H, Roy JS, Dumoulin C. Effects of radiation therapy on the structure and function of the pelvic floor muscles of patients with cancer in the pelvic area: a systematic review. J Cancer Surviv. 2016 Apr;10(2):351-62. PMID: WOS:000373079800014. doi: 10.1007/s11764-015-0481-8.
13. Bernard S, Moffet H, Plante M, Ouellet MP, Leblond J, Dumoulin C. Pelvic-Floor Properties in Women Reporting Urinary Incontinence After Surgery and Radiotherapy for Endometrial Cancer. Phys Ther. 2017 Apr 1;97(4):438-48. PMID: 28201796. doi: 10.1093/ptj/pzx012.
14. Yang EJ, Lim JY, Rah UW, Kim YB. Effect of a pelvic floor muscle training program on gynecologic cancer survivors with pelvic floor dysfunction: A randomized controlled trial. Gynecologic Oncology. 2012 Jun;125(3):705-11. PMID: WOS:000304745100040. doi: 10.1016/j.ygyno.2012.03.045.
15. Rutledge TL, Rogers R, Lee SJ, Muller CY. A pilot randomized control trial to evaluate pelvic floor muscle training for urinary incontinence among gynecologic cancer survivors. Gynecologic Oncology. 2014 Jan;132(1):154-8. PMID: WOS:000330912800029. doi: 10.1016/j.ygyno.2013.10.024.
16. Brennen R, Lin K, Denehy L, Frawley H. The effect of pelvic floor muscle interventions on pelvic floor dysfunction after gynaecological cancer treatment: A systematic review. Physical Therapy. 2020;100(8):1357–71. doi: https://doi.org/10.1093/ptj/pzaa081.

17. Cancer Australia. National Framework for Gynaecological Cancer Control. Surry Hills, NSW: Cancer Australia; 2016.
18. Nakayama N, Tsuji T, Aoyama M, Fujino T, Liu MG. Quality of life and the prevalence of urinary incontinence after surgical treatment for gynecologic cancer: a questionnaire survey. Bmc Womens Health. 2020 Jul;20(1). PMID: WOS:000552395900001. doi: 10.1186/s12905-020-01012-7.
19. Crean-Tate KK, Faubion SS, Pederson HJ, Vencill JA, Batur P. Management of genitourinary syndrome of menopause in female cancer patients: a focus on vaginal hormonal therapy. American Journal of Obstetrics & Gynecology. 2020;222(2):103-13. doi: 10.1016/j.ajog.2019.08.043.
20. Faubion SS, MacLaughlin KL, Long ME, Pruthi S, Casey PM. Surveillance and Care of the Gynecologic Cancer Survivor. J Womens Health (Larchmt). 2015;24(11):899-906. PMID: 26208166. doi: 10.1089/jwh.2014.5127.
21. Brennen R, K L, L D, S S, H F. A whole 'nother layer of difficulty…: Patient and clinician experiences of pelvic floor dysfunction after gynaecological cancer. International Continence Society Conference Abstract [Internet]. 2021. Available from: https://www.ics.org/2021/abstract/509.
22. Huang ZH, Wu SY, Yu T, Hu AL. Efficacy of telemedicine for urinary incontinence in women: a systematic review and meta-analysis of randomized controlled trials. International Urogynecology Journal. 2020;31:1507-13. PMID: WOS:000536736400001. doi: 10.1007/s00192-020-04340-2.
23. Salmon VE, Hay-Smith EJC, Jarvie R, Dean S, Terry R, Frawley H, et al. Implementing pelvic floor muscle training in women's childbearing years: A critical interpretive synthesis of individual, professional, and service issues. Neurourology and Urodynamics. 2020 Feb;39(2):863-70. PMID: WOS:000502938800001. doi: 10.1002/nau.24256.
24. Bump RC, Hurt WG, Fantl JA, Wyman JF. Assessment of Kegel pelvic muscle exercise performance after brief verbal instruction. American Journal of Obstetrics and Gynecology. 1991;165(2):322-7; discussion 7-9.
25. Mateus-Vasconcelos ECL, Brito LGO, Driusso P, Silva TD, Antonio FI, Ferreira CHJ. Effects of three interventions in facilitating voluntary pelvic floor muscle contraction in women: a randomized controlled trial. Brazilian Journal of Physical Therapy. 2018 Sep-Oct;22(5):391-9. PMID: WOS:000445545500007. doi: 10.1016/j.bjpt.2017.12.006.
26. Cacciari LP, Amorim AC, Passaro AC, Dumoulin C, Sacco ICN. Intravaginal pressure profile of continent and incontinent women. Journal of Biomechanics. 2020 Jan;99. PMID: WOS:000513294600040. doi: 10.1016/j.jbiomech.2019.109572.
27. Cacciari LP, Kruger J, Goodman J, Budgett D, Dumoulin C. Reliability and validity of intravaginal pressure measurements with a new intravaginal pressure device: The FemFit (R). Neurourology and Urodynamics. 2020 Jan;39(1):253-60. PMID: WOS:000506930700027. doi: 10.1002/nau.24179.
28. Mateus-Vasconcelos ECL, Ribeiro AM, Antonio FI, Brito LGD, Ferreira CHJ. Physiotherapy methods to facilitate pelvic floor muscle contraction: A systematic review. Physiotherapy Theory and Practice. 2018;34(6):420-32. PMID: WOS:000427278100002. doi: 10.1080/09593985.2017.1419520.
29. Dumoulin C, Cacciari LP, Hay-Smith EJC. Pelvic floor muscle training versus no treatment, or inactive control treatments, for urinary incontinence in women. Cochrane Database Sys Rev. 2018 Oct 4;10:Cd005654. PMID: 30288727. doi: 10.1002/14651858.CD005654.pub4.
30. Lin KY, Edbrooke L, Granger CL, Denehy L, Frawley HC. The impact of gynaecological cancer treatment on physical activity levels: a systematic review of observational studies. Braz J Phys Ther. 2019 Mar-Apr;23(2):79-92. PMID: WOS:000461964200002. doi: 10.1016/j.bjpt.2018.11.007.
31. Dumoulin C, Hay-Smith J, Frawley H, McClurg D, Alewijnse D, Bo K, et al. 2014 consensus statement on improving pelvic floor muscle training adherence: International Continence Society 2011 State-of-the-Science Seminar. Neurourol Urodyn. 2015 Sep;34(7):600-5. PMID: 25998603. doi: 10.1002/nau.22796.
32. Slade SC, Hay-Smith J, Mastwyk S, Morris ME, Frawley H. Strategies to assist uptake of pelvic floor muscle training for people with urinary incontinence: A clinician viewpoint. Neurourology and Urodynamics. 2018 Nov;37(8):2658-68. PMID: WOS:000448184900044. doi: 10.1002/nau.23716.

33. Frawley HC, Dean SG, Slade SC, Hay-Smith EJC. Is Pelvic-Floor Muscle Training a Physical Therapy or a Behavioral Therapy? A Call to Name and Report the Physical, Cognitive, and Behavioral Elements. Phys Ther. 2017 Apr 1;97(4):425-37. PMID: 28499001. doi: 10.1093/ptj/pzx006.
34. Avery K, Donovan J, Peters TJ, Shaw C, Gotoh M, Abrams P. ICIQ: A brief and robust measure for evaluating the symptoms and impact of urinary incontinence. Neurourology and Urodynamics. 2004;23(4):322-30. PMID: ISI_WOS_XML.
35. Nystrom E, Sjostrom M, Stenlund H, Samuelsson E. ICIQ symptom and quality of life instruments measure clinically relevant improvements in women with stress urinary incontinence. Neurourology and Urodynamics. 2015 Nov;34(8):747-51. PMID: WOS:000362963200008. doi: 10.1002/nau.22657.
36. Landes SJ, McBain SA, Curran GM. An introduction to effectiveness-implementation hybrid designs. Psychiatry Research. 2019 2019/10/01/;280:112513. doi: https://doi.org/10.1016/j.psychres.2019.112513.
37. Chan AW, Tetzlaff JM, Altman DG, Laupacis A, Gotzsche PC, Krle AJK, et al. SPIRIT 2013 Statement: defining standard protocol items for clinical trials. Rev Panam Salud Publica. 2015 Dec;38(6):506-14. PMID: 27440100.
38. Boutron I, Moher D, Altman DG, Schulz KF, Ravaud P. Extending the CONSORT statement to randomized trials of nonpharmacologic treatment: explanation and elaboration. Annals of internal medicine. 2008 Feb 19;148(4):295-309. PMID: 18283207. doi: 10.7326/0003-4819-148-4-200802190-00008.
39. Moher D, Hopewell S, Schulz KF, Montori V, Gotzsche PC, Devereaux PJ, et al. CONSORT 2010 explanation and elaboration: updated guidelines for reporting parallel group randomised trials. International journal of surgery (London, England). 2012;10(1):28-55. PMID: 22036893. doi: 10.1016/j.ijsu.2011.10.001.
40. Zwarenstein M, Treweek S, Gagnier JJ, Altman DG, Tunis S, Haynes B, et al. Improving the reporting of pragmatic trials: an extension of the CONSORT statement. BMJ (Clinical research ed). 2008 Nov 11;337:a2390. PMID: 19001484. doi: 10.1136/bmj.a2390.
41. Slade SC, Morris ME, Frawley H, Hay-Smith J. Comprehensive reporting of pelvic floor muscle training for urinary incontinence: CERT-PFMT. Physiotherapy. 2021 Mar 6;112:103-12. PMID: 34062452. doi: 10.1016/j.physio.2021.03.001.
42. Kelleher CJ, Cardozo LD, Khullar V, Salvatore S. A new questionnaire to assess the quality of life of urinary incontinent women. Br J Obstet Gynaecol. 1997 Dec;104(12):1374-9. PMID: 9422015. doi: 10.1111/j.1471-0528.1997.tb11006.x.
43. Peterson TV, Karp DR, Aguilar VC, Davila GW. Validation of a global pelvic floor symptom bother questionnaire. International Urogynecology Journal. 2010 2010/09/01;21(9):1129-35. doi: 10.1007/s00192-010-1148-7.
44. Rabin R, de Charro F. EQ-5D: a measure of health status from the EuroQol Group. Ann Med. 2001 Jul;33(5):337-43. PMID: 11491192. doi: 10.3109/07853890109002087.
45. Foundation ER. EQ-5D-5L user guide. [cited 2021 July]; Available from: https://euroqol.org/publications/user-guides/.
46. Tavares J, Oliveira T. Electronic Health Record Patient Portal Adoption by Health Care Consumers: An Acceptance Model and Survey. J Med Internet Res. 2016 Mar 2;18(3):e49. PMID: 26935646. doi: 10.2196/jmir.5069.
47. Venkatesh V, Thong JYL, Xu X. Consumer Acceptance and Use of Information Technology: Extending the Unified Theory of Acceptance and Use of Technology. MIS Quarterly. 2012;36(1):157-78. doi: 10.2307/41410412.
48. Mäder U, Martin BW, Schutz Y, Marti B. Validity of four short physical activity questionnaires in middle-aged persons. Med Sci Sports Exerc. 2006 Jul;38(7):1255-66. PMID: 16826022. doi: 10.1249/01.mss.0000227310.18902.28.

49. Sangha O, Stucki G, Liang MH, Fossel AH, Katz JN. The Self-Administered Comorbidity Questionnaire: a new method to assess comorbidity for clinical and health services research. Arthritis Rheum. 2003 Apr 15;49(2):156-63. PMID: 12687505. doi: 10.1002/art.10993.
50. Dowell CJ, Bryant CM, Moore KH, Simons AM. Calculating the direct costs of urinary incontinence: a new test instrument. BJU Int. 1999 Apr;83(6):596-606. PMID: 10233564. doi: 10.1046/j.1464-410x.1999.00993.x.
51. Frawley HC, Phillips BA, Bø K, Galea MP. Physiotherapy as an adjunct to prolapse surgery: an assessor-blinded randomized controlled trial. Neurourol Urodyn. 2010 Jun;29(5):719-25. PMID: 19816918. doi: 10.1002/nau.20828.
52. Frawley HC, Hagen S, Sherburn M, Neumann P, Hay-Smith J, Herbison P, et al. Changes in prolapse following pelvic floor muscle training: a randomised controlled trial. Neurourol Urodyn. 2012;31:938-39.
53. Administration TG. The Australian Register of Therapeutic Goods. [cited 2021 15th July]; Available from: https://tga-search.clients.funnelback.com/s/search.html?collection=tga-artg&profile=record&meta_i=345787.
54. Dumoulin C, Morin M, Danieli C, Cacciari LP, Mayrand M, Tousignant M, et al. Group-Based vs Individual Pelvic Floor Muscle Training to Treat Urinary Incontinence in Older Women: A Randomized Clinical Trial. JAMA Intern Med. 2020;Published online August 3, 2020. doi: doi:10.1001/jamainternmed.2020.2993.
55. Council NHaMR. Guidance: Safety monitoring and reporting in clinical trials involving therapeutic goods. Canberra: National Health and Medical Research Council, 2016.
56. Drummond MF, Sculpher MJ, Claxton K, Stoddart GL, Torrance GW. Methods for the Economic Evaluation of Health Care Programmes. Oxford: Oxford: Oxford University Press; 2015. ISBN: 9780199665884.
57. National Cancer Institute. (2017). Common Terminology Criteria for Adverse Events (CTCAE) Version 5.0. Availble from: https://ctep.cancer.gov/protocolDevelopment/electronic_applications/docs/CTCAE_v5_Quick_Reference_5x7.pdf
58. National Cancer Institute. (2013). NCI Guidelines for Investigators: Adverse Event Reporting Requirements for DCTD (CTEP and CIP) and DCP INDs and IDEs. Avaialble from: https://ctep.cancer.gov/protocolDevelopment/electronic_applications/docs/aeguidelines.pdf
