# Supplementary material for: Telehealth exercise for continence after gynaecological cancer treatment (TELE-CONNECT): a protocol for a co-designed pragmatic randomised controlled trial
Source: BMC Womens Health. 2024 Sep 27;24:529. doi: 10.1186/s12905-024-03365-9 (PMC11430120; doi:10.1186/s12905-024-03365-9)
Supplement: Supplementary file 2 — Additional file 2: TELE-CONNECT study participant information form. [file 12905_2024_3365_MOESM2_ESM.pdf]

Participant Information Sheet

INSERT SITE NAME

**Title**	Effectiveness of telehealth-delivered exercise to treat incontinence in women following gynaecological cancer: a co-designed pragmatic randomised controlled trial
**Short Title**	TELE-CONNECT: **Tel**ehealth **E**xercise for **Con**tinence After Gy**n**aecological **C**ancer **T**reatment
**Project Sponsor**	The University of Melbourne
**Principal Investigator**	Associate Professor Helena Frawley
**Associate Investigator**	Prof Linda Denehy, Prof Kim Bennell, Dr Rachel Nelligan, Angela Ravi, A/Prof Orla McNally, A/Prof Simon Hyde, Dr Shih-Ern Yao, A/Prof Karen Lamb, Fiona McManus, Nipuni Susanto, Dr Zobaida Edib, Peixuan Li
**Location**	*INSERT SITE NAME*

Part 1 What does my participation involve?

1 Introduction

You are invited to take part in this research project conducted by researchers at the University of Melbourne. This is because you have had treatment for gynaecological cancer. This research project is investigating treatments for bladder leakage (urinary incontinence) in women who have undergone treatment for Stage I, II or III uterine, cervical, fallopian tube, peritoneal or ovarian cancer or borderline ovarian tumour. The treatment we are evaluating is pelvic floor physiotherapy delivered remotely using video-calls (Zoom or other software). The pelvic floor supports your pelvic organs (bladder, vagina and bowel), and pelvic floor physiotherapy teaches women exercises for their pelvic floor muscles, to help support and control the pelvic organs. We are comparing this to the usual care women receive for urinary incontinence after gynaecological cancer treatment.

This Participant Information Sheet/Consent Form tells you about the research project. It explains the tests and research involved. Knowing what is involved will help you decide if you want to take part in the research or not.

Please read this information carefully. Ask questions about anything that you don't understand or want to know more about. Before deciding whether or not to take part, you might want to talk about it with a relative, friend or local doctor.

Participation in this research is voluntary. If you don't wish to take part, you don't have to. You will receive your usual healthcare whether or not you take part.

If you decide you want to take part in the research project, you will be asked to sign the consent section. By signing it you are telling us that you:
• Understand what you have read
• Consent to take part in the research project
• Consent to the tests and treatments that are described
• Consent to the use of your personal and health information as described.

You will be given a copy of this Participant Information and Consent Form to keep.

2 What is the purpose of this research?

This study will help us to better understand treatments that may help women experiencing urinary incontinence after having treatment for gynaecological cancer. Urinary incontinence is common in women, especially women who have undergone treatment for gynaecological cancer.

Pelvic floor physiotherapy has been used successfully for urinary incontinence in women who have not had gynaecological cancer, but it has not been extensively tested in women who have had gynaecological cancer. Also, many women find it challenging to visit a pelvic floor physiotherapist regularly for treatment. Delivering pelvic floor physiotherapy over video-calls may be one way to improve access and provide treatment to women with urinary incontinence after gynaecological cancer treatment. Delivering pelvic floor physiotherapy over video-calls has not been tested in women with urinary incontinence following treatment for gynaecological cancer. Therefore, the aim of this research project is to test a new treatment for women with urinary incontinence after gynaecological cancer treatment - pelvic floor physiotherapy delivered via video-calls. As no other research has tested this yet, it is important that we do this study.

Ultimately, this research aims to reduce the impact of gynaecological cancer treatment on bladder problems and improve the quality of life of women who have had gynaecological cancer treatment.

This research is led by Associate Professor Helena Frawley and has been funded by the Victorian Cancer Agency.

3 What does participation in this research involve and what will I be asked to do?

The best way to test the effectiveness of this new treatment is to compare it to the usual care women receive for urinary incontinence, after they finish gynaecological cancer treatment. Therefore, if you take part in this study, you will be asked to participate in **one** of two different treatment groups 1) usual care or 2) pelvic floor physiotherapy delivered via video-calls. The reason we are comparing these two treatments is because we do not know which treatment is best for treating urinary incontinence after gynaecological cancer treatment. The results of this study will tell us if one treatment is better or if they are the same. Please read Steps 1 to 4 below to find out what the research involves and what you will be asked to do.

Step 1: Screening

If you consent to be contacted by the research team, a member of the research team will telephone you. Or, if a member of the research team is onsite during your appointment with your gynaecology team and you consent to talk to them on the day, they may speak with you in person. During this conversation they will discuss the study in more detail and ask you some questions to confirm if you are eligible to participate. This conversation will take around 10-20 minutes. If you are interested in taking part and are eligible they will send you a copy of this document to read.

You can speak to a member of the research team about the study at any stage of your cancer treatment. However, you will only be able to participate once you have finished your primary gynaecological cancer treatment 6 months ago or adjuvant therapy completed at least 3 months ago , to give you time to recover from your cancer treatments. If you do speak to the research team during your cancer treatment or within 6 months of your cancer treatment finishing, and you are interested in being involved in this research, the research team will schedule a follow-up phone call. The follow-up call will be at a date that is 6 months after your completed cancer treatment.

Step 2: Baseline assessment

Once it is 6 months after your cancer treatment has finished, if you are eligible, and if you decide to take part, you will be asked to complete a consent form. The consent form is included within this document. It can be completed on paper and returned to the research team via post, or you can request that the researcher emails you a link so that you can complete it via a secure online platform.

After completing the consent form, you will be asked to complete a questionnaire about yourself. This questionnaire will ask you information about yourself, your general health, medical history including cancer treatments, bladder symptoms, quality of life and your thoughts about videoconferencing (video-calls). You will also be asked to record any bladder leakage you experience over a 7-day period, in a diary called an 'Accident Diary'. The purpose of this questionnaire and the Accident Diary is to record how your bladder symptoms affect you before you start the study. Answering the questions can take some time (up to 30-45 minutes to complete). The questionnaire can be completed online or with pen and paper and returned via post, depending on your preference. You'll be asked to return the completed Accident Diary to the research team via post or by scanning and emailing it. If you decide to participate in this study, your doctor or nurse may provide specific information from your medical record to the research team about your cancer diagnosis and treatment. They will not provide any information that is not directly relevant to this study.

Step 3: Allocation to a study treatment.

If you decide to take part, you will be participating in a randomised controlled research project. Once the research team receive your completed consent form, baseline questionnaire, and 7-day Accident Diary a member of the research team will call you. During this call they will allocate you to one of the two treatment groups in this study. To try to make sure the groups are similar, each participant is put into a group by chance (randomly) using a computer program. In this research project you will have an equal chance of being put into either of the two groups. You won't be able to choose or change groups along the way. There are no costs associated with participating in this research project or either of the treatment groups.

Group 1: Usual Care

If you are allocated to Group 1:

- You will be sent high quality bladder and bowel advice handouts via email or post (dependent on your preference)
- You will also receive one, 15-minute telephone call with a female, qualified and experienced physiotherapist. This will be scheduled by the research team within 1-2 weeks of the handouts being sent to you and will be at a time that suits you. During this consultation, the physiotherapist will check you have received the handouts and answer any handout or study related questions you might have.

Group 2: pelvic floor physiotherapy delivered via video-calls

If you are allocated to Group 2:

- You will receive high quality bladder and bowel advice handouts. You will also receive information about having video-call consultations and guidance on how to use videoconference software (Zoom or other platform) for these consultations. This information will be sent via post or email depending on your preference. In addition, a member of the research team will be available to have a practice videoconference consultation with you, to ensure you are able to connect successfully for your consultations with the physiotherapist.
- You will also receive your own pelvic floor muscle biofeedback device, via post.
- The research team will schedule your first physiotherapy consultation for you and will send you a reminder mobile phone text message 24-hours before this scheduled appointment. The physiotherapist will schedule any remaining consultations with you later. Consultations will be approximately 2 weeks apart and will be at a time that suits you and in accordance with the physiotherapist's availability. Each consultation will be 30-60 minutes long.
- Over 16-weeks you will have a total of 8 video-call consultations with the same pelvic floor physiotherapist. The consultations will involve the physiotherapist teaching you pelvic floor muscle exercises and other strategies to assist with good bladder and bowel function. During your consultations you will be asked to use a small pelvic floor biofeedback sensor, a soft and flexible object made of silicone, the size of your index finger, that you put into your vagina. The sensor will help you identify your muscles, help with your exercises and help the physiotherapist review your progress. The physiotherapist will give you exercises using your femfit app and other activities to do at home in between appointments. The physiotherapist will record brief clinical notes about each of their consultations with you. The physiotherapist will also be able to remotely access information about your pelvic floor muscle exercises from your biofeedback device. This

will be possible through a smartphone 'app' that works with your sensor. This information will be used as part of the analysis in this study.

- You will also be asked to keep a record of any pelvic floor muscle exercises you perform within a smartphone app during the study.
- If you consent, three of your consultations with the physiotherapist will be recorded. Only audio recordings will be provided to the research team. A member of the research team (a female physiotherapist) will review audio recordings to monitor that the consultations are being delivered as planned by the study physiotherapist.
- Women in Group 2 will also be able to keep their pelvic floor biofeedback device. If you incur any costs associated with your participation (e.g. purchase of a webcam) you will be reimbursed the monetary value via a gift card.

<u>Step 4: Follow-up assessments</u>

After you have been involved in the research for 17 weeks, you will be asked to complete a second questionnaire. We will ask you to complete a third and final questionnaire 52 weeks after you have been involved in the research. Both the 17-week and 52-week questionnaires will take you around 15-25 minutes to complete. The questionnaires can be completed online, where the research team will email you a link to complete the questionnaire on a secure online platform or using paper and pen, where the questionnaire will be posted out to you and returned via post in a pre-paid envelope.

There are no costs associated with participating in this research project, nor will you be paid. You will receive a $20 gift card in appreciation of your time on completion of the third and final study questionnaire.

This research project has been designed to make sure the researchers interpret the results in a fair and appropriate way and avoids researchers or participants jumping to conclusions.

4 Other relevant information about the research project

This research project will involve 72 women who have received gynaecological cancer treatment. There will be 36 women allocated to each study group. Victorian hospital sites include Monash Health, Western Health, Mercy Hospital for Women, the Royal Women's Hospital, participating private hospitals and women recruited from the community. This research involves researchers and clinicians from The University of Melbourne, and from the hospital sites working in collaboration with us, as well as consumers who contribute their experience and perspectives of having undergone treatment for gynaecological cancer. In addition, researchers from the University of Montreal and The University of Auckland are also part of the broader study team and have provided input into the design of this research. They will not be involved in the running of this study nor in any analysis of the data. A member of the study team from The University of Auckland, Dr Jennifer Kruger, is the inventor of the biofeedback device used in this study and is the CEO of the company that distributes the device. Dr Kruger will not be involved in the running of this study nor in any analysis of the data.

5 Do I have to take part in this research project?

Participation in any research project is voluntary. If you do not wish to take part, you do not have to. If you decide to take part and later change your mind, you are free to withdraw from the project at any stage. If you do decide to take part, you will be given this Participant Information and the Consent Form to sign and you will be given a copy to keep. Your decision whether to take part or not to take part, or to take part and then withdraw, will not affect your routine treatment, your relationship with those treating you. or your relationship with *INSERT SITE NAME*.

6 What are the alternatives to participation?

Your participation in this research project is voluntary. Normal care will be continued in the event you choose not to participate. No standard treatment will be withheld at any stage. You do not have to take part in this research project to receive treatment for pelvic floor problems at *INSERT SITE NAME*. Other options are available; these include seeking a referral from your treating doctors and nurses to the women's health physiotherapy team at the site you had your cancer treatment. You can also discuss your options with your local doctor.

7 What are the possible benefits of taking part?

We cannot guarantee or promise that you will receive any benefits from this research. Possible benefits may include that you receive useful information about bladder and bowel health, and you may possibly experience some improvement in your bladder leakage.

The results of this study will help us to understand if video-call delivered pelvic floor physiotherapy is beneficial for women who have had gynaecological cancer treatment, or not. This is an important step towards developing effective treatments to help women with bladder problems after gynaecological cancer treatment. Your participation in this study is potentially valuable to future women.

8 What are the possible risks and disadvantages of taking part?

We do not foresee any risks or disadvantages to your participation in this project. If you become upset or distressed because of your involvement in any part of this research (e.g., from receiving an invitation to participate in this study, completing the study questionnaires or your actual participation in the study) please contact the Principal Investigator Dr Helena Frawley on 0418584813. The Principal Investigator will discuss your concerns and make an appropriate plan to support you. If you become distressed during the course of the project, you will be offered an option to pause and continue after a short break or reschedule to another time. You will also be provided with the option to cease your involvement with the project completely. In addition, you may choose to suspend or end your participation in the research project. You may also contact BeyondBlue (1300 22 4636) and LifeLine (13 11 14) to seek any advice.

After menopause and after gynaecological cancer treatment, some women may find inserting an object into the vagina is uncomfortable, and some women may experience vaginal bleeding. This scenario would be unusual, however this may be the case with this intra-vaginal biofeedback sensor, although the sensor is smaller than an index finger and smaller than an intra-vaginal dilator you may have used. This is unlikely to be severe and may resolve as you become accustomed to using it, or you may cease using it and continue in the study without using the device. You can discuss any concerns about this with your physiotherapist or the Principal Investigator. During your consultations, your physiotherapist will ask you if you have any discomfort using the device and modify your treatment accordingly.

The pelvic floor biofeedback device used in study Group 2 is a low-risk device which has undergone extensive electro-magnetic compatibility, biocompatibility and electrical safety testing. The device is registered with the Australian Therapeutic Goods Administration, is approved for use in Australia and is commercially available.

9 What if new information arises during this research project?

Sometimes during the course of a research project, new information becomes available about the treatments that are being studied. If this happens, a member of the research team will tell you about it and discuss with you whether you want to continue in the research project. If you decide to withdraw, please let the research team know. If you later decide to continue in the research project you will be asked to sign an updated consent form. Also, on receiving new information, the research team might consider it to be in your best interests to withdraw you from the research project. If this happens, they will explain the reasons.

10 Can I have other treatments during this research project?

Whilst you are participating in this research project, you are able to take any medications you have been taking. We do ask that you do not see a physiotherapist for treatment of bladder problems other than the physiotherapist who contacts you as part of this research during the first 17 weeks you are involved in this study.

11 What if I withdraw from this research project?

If you decide to withdraw from this research project, please notify a member of the research team before you withdraw. A member of the research team will inform you if there are any special requirements linked to withdrawing. If you do withdraw your consent during the research project, the study staff will not collect additional personal information from you, although personal information already collected will be retained to ensure that the results of the research project can be measured properly and to comply with law. You should be aware that data collected by the University of

Melbourne up to the time you withdraw will form part of the research project results. If you do not want them to do this, you must tell them before you join the research project.

12 Could this research project be stopped unexpectedly?

Although it is unlikely, if this project is terminated before its completion, we will notify you. You will continue to be provided with standard care for your medical condition.

13 What happens when the research project ends?

We will provide a summary of the findings to all participants who are interested in the results. You can request to receive the summary of findings once the study has concluded, and we will post or email this to you when they are available.

Part 2 How is the research project being conducted?

14 What will happen to information about me?

By signing the consent form, you consent to the relevant research staff collecting and using personal information about you for the research project. Any information obtained in connection with this research project that can identify you will remain confidential. Your name and any identifying details will be removed before entry into the study database and no identifiable information will be stored. A number code will be assigned to you, and only this coded number will be used during the study to identify your information. The study code will not be reported. Hard copies of questionnaires will be scanned and original hard copies will be stored in a locked filing cabinet in a locked room for 15 years after the study has finished. At the end of the storage period (fifteen years), data will be shredded and disposed of using the health service confidential documents disposal system. The electronic copies of questionnaires, along with any electronically recorded clinical assessment data (i.e. pelvic floor muscle exercise data from the biofeedback device if you are allocated to Group 2), will be stored on a password-protected computer and kept in locked facilities in the office of the Principal Investigator. Access to the records will be by the research team only. Data will be kept for 15 years as per Australian guidelines. Before the details from this study are destroyed, if you provide your consent, details will be entered into a databank. This is optional. The information entered into the databank will not be able to be linked to an individual participant in this study. Information in the databank will be accessed by this research team or future research teams who undertake research related to pelvic floor function in women. You can choose to opt in or out of your data from this study being entered into the databank by selecting your preferred response on the “Additional Agreements” section of the Consent Form.

If you are allocated by chance to the group receiving video calls from the physiotherapist you will be asked to use a pelvic floor muscle biofeedback device (as described on page 3 under Group 2). This device displays and records information about your pelvic floor muscle exercises through a mobile phone app. The app will collect information (data) about your pelvic floor exercises, each time you use the app. You will be asked to download this app onto your mobile phone. This will involve reading and agreeing to the terms of use of the app, similar to downloading other apps onto your phone, like the Medicare app, MyGov app, etc. Both the device and the app have been developed by pelvic floor researchers in New Zealand. Data from the device and app is transmitted via a wifi connection to an independent third party, a company based in New Zealand. This third party securely stores the data that is collected when the app is used. For the purposes of this study, the researchers in this study will access the data stored in the app. They will do this by downloading your data directly from the third party’s online server in New Zealand. Any information that could identify you will be removed and replaced with a unique study code. Only data from the app with unique study codes will be stored in the secure password protected database for this study. Data will be downloaded from the app solely for the purposes of this study, as described on page 3.

Your information will only be used for the purpose of this research project or future research you may consent to, and it will only be disclosed with your permission, except as required by law.

In addition, information about you may be obtained from your health records held at *[INSERT SITE NAME]*, for the purpose of this research. By signing the consent form you agree to the research team accessing your health records if they are relevant to your participation in this research project.

It is anticipated that the results of this research project will be published and/or presented in a variety of forums. In any publication and/or presentation, information will be provided in such a way that you cannot be identified, except with your permission. Only non-identifiable summary, i.e. not individual, results will be publicly reported.

In accordance with relevant Australian and/or Victorian privacy and other relevant laws, you have the right to request access to the information collected and stored by the research team about you. You also have the right to request that any information with which you disagree be corrected. Please contact the research team member named at the end of this document if you would like to access your information. Any information obtained for the purpose of this research project and for the future research described above that can identify you will be treated as confidential and securely stored. It will be disclosed only with your permission, or as required by law.

15 Complaints and compensation

If you suffer any injuries or complications as a result of this research project, you should contact the research team as soon as possible and you will be assisted with arranging appropriate medical treatment. If you are eligible for Medicare, you can receive any medical treatment required to treat the injury or complication, free of charge, as a public patient in any Australian public hospital.

16 Who is organising and funding the research?

This research project is being conducted by Associate Professor Helena Frawley, at the University of Melbourne. It is funded by the Victorian Cancer Agency.

17 Who has reviewed the research project?

All research in Australia involving humans is reviewed by an independent group of people called a Human Research Ethics Committee (HREC). The ethical aspects of this research project have been approved by the HREC of Monash Health. This project will be carried out according to the *National Statement on Ethical Conduct in Human Research (2018)*. This statement has been developed to protect the interests of people who agree to participate in human research studies.

18 Further information and who to contact

If you want any further information concerning this project or if you have any medical problems which may be related to your involvement in the project (for example, any side effects), you can contact the principal study physiotherapist A/Prof Helena Frawley on 0418 584813.

If you have any complaints about any aspect of the project, the way it is being conducted or any questions about being a research participant in general, then you may contact:

Complaints contact person

Name	HREC Executive Officer
Position	HREC Executive Officer
Telephone	(03) 9594 4611
Email	research@monashhealth.org

Reviewing HREC approving this research and HREC Executive Officer details

Reviewing HREC name	Monash Health Human Research Ethics Committee
HREC Executive Officer	HREC Executive Officer
Telephone	(03) 9594 4611
Email	research@monashhealth.org

Local HREC Office contact (Single Site - Research Governance Officer)

Name	HREC Executive Officer
Position	HREC Executive Officer
Telephone	(03) 9594 4611
Email	research@monashhealth.org
